# Anchor Engineering of Hole‐Selective Enamine‐Based Self‐Assembling Monolayers for Perovskite Solar Cells

**DOI:** 10.1002/advs.76950

**Published:** 2026-08-21

**Authors:** Deimante Krisiune, Yuxuan Yang, Yongde Xu, Hao Tian, Chuanxiao Xiao, Jianxing Xia, Mahan Saberi Zafarghandi, Ali Keshavarz Mohammadian, Negar Ashari Astani, Kasparas Rakstys, Yi Zhang, Vytautas Getautis

**Affiliations:** ^1^ Department of Organic Chemistry Kaunas University of Technology Kaunas Lithuania; ^2^ Institute of Molecular Plus School of Chemical Engineering and Technology Tianjin University Tianjin China; ^3^ Ningbo Institute of Materials Technology and Engineering Chinese Academy of Sciences Ningbo China; ^4^ Department of Physics and Energy Engineering Amirkabir University of Technology Tehran Iran

**Keywords:** enamine, energy conversion efficiency, materials science, monolayer, nanotechnology, perovskite, photovoltaic system, renewable energy, solar energy

## Abstract

The transition to renewable energy sources, particularly solar power, has highlighted the potential of next‐generation perovskite solar cells (PSCs), which have achieved a power conversion efficiency (PCE) over 27%, rivaling conventional silicon solar cells. A critical component in p‐i‐n PSCs is hole transporting layer (HTL), where polymers like PTAA have shown promise but face challenges in efficiency and commercial viability hindered by high costs and complex synthesis. Recently, enamine‐based HTMs have emerged as a promising alternative due to their superior charge transport properties, structure's tunability, and cost‐effectiveness. Additionally, self‐assembling monolayers (SAMs) have been explored to improve inverted PSC performance by enhancing interface properties and reducing material use. This study combines enamine chemistry and self‐assembly to engineer enamine‐based SAMs with various structural units having ─COOH and ─PO(OH)_2_ anchoring groups to optimize SAM/TCO interface and reduce recombination losses. The resulting p‐i‐n devices exhibit high power conversion efficiencies (>25.5%) and improved stability. The champion mini‐module with an aperture area of 29.7 cm^2^ realizes a PCE of 23.14% with an FF of 83.11%, highlighting the potential of these materials for scalable photovoltaic applications.

## Graphical abstract

This work presents novel enamine‐based self‐assembling monolayers with engineered anchoring groups as hole‐selective contacts for inverted perovskite solar cells. The conjugated molecular design suppresses interfacial defects, optimizes charge extraction, and prevents aggregation. Consequently, the engineered devices achieve outstanding power conversion efficiencies exceeding 25.5% and demonstrate robust long‐term stability, advancing scalable photovoltaic applications.

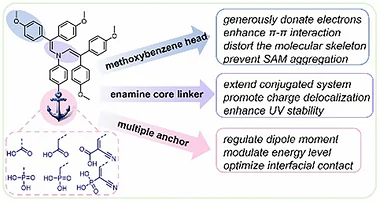

## 1 Introduction

As the world shifts towards renewable energy sources, the utilization of solar energy is inevitable. Among the most promising methods to harness solar power are next‐generation perovskite solar cells (PSCs), which have achieved a power conversion efficiency (PCE) of 26.7 % [1], equivalent to the record certified for conventional crystalline silicon solar cells (∼27 %) [2]. A significant advantage associated with PSCs is low‐cost fabrication [3, 4], enabling scalable manufacturing through solution‐based processes such as spin‐coating [5, 6] and inkjet printing [7, 8]. Furthermore, the versatility of the perovskite material allows the development of flexible, lightweight, and potentially transparent solar cells, expanding their potential applications [9, 10, 11].

In general, regular (n‐i‐p) PSCs demonstrated higher efficiency, though lately devices with inverted (p‐i‐n) architecture have attracted more interest mainly because of better long‐term stability [12, 13, 14], since doping is unnecessary for charge selective contacts in PSCs with such structure. Additionally, p‐i‐n devices have several other attractive advantages, including low‐temperature processability [15, 16, 17] and considerably less noticeable hysteresis [18, 19]. Lastly, inverted PSCs show great potential for the construction of perovskite‐based tandem solar cells due to the reduced parasitic absorption [20, 21].

A crucial component for PSCs fabrication is hole transporting layer (HTL), which is responsible for collecting holes at the perovskite/HTL interface while ensuring minimal energy loss and maintaining long‐term stability under operating conditions. Relatively recently, polymers have dominated as HTLs for inverted PSCs, primarily because of their superior resistivity to perovskite precursors, and PTAA (poly [bis(4‐phenyl)(2,4,6‐trimethylphenyl)amine) can be considered as one of the most efficacious polymeric materials, allowing p‐i‐n devices to exhibit almost 24% efficiency [22, 23, 24]. Another conductive polymers commonly exploited in inverted PSCs are PEDOT:PSS [25], poly‐TPD [26] and P3HT [27], yet it is challenging to fabricate the highest efficiency devices with such materials mainly due to the poor energy level alignment and large recombination losses [28, 29, 30]. Additionally, their commercial viability is limited due to the difficult synthesis procedures, poor reproducibility, and extremely high cost [31, 32, 33].

Molecular enamine‐based HTLs have arisen as another potential candidate for n‐i‐p PSCs in 2017 [34] due to their unique chemical structure and excellent charge transporting properties. These materials, characterized by their nitrogen‐rich motifs and electron‐donating properties, offer significant advantages in terms of high hole mobility, good energy level alignment with perovskite absorbers, tunable chemical structures, and the possibility for low‐cost solution processing [35, 36, 37, 38]. Furthermore, enamine condensation chemistry has a number of benefits over transition metal‐catalyzed reaction, including the elimination of costly catalysts and a straightforward product purification procedure, substantially decreasing the final product's cost [39, 40].

Several years ago, self‐assembling monolayers (SAMs) emerged as a promising alternative in the development of inverted PSCs, offering a versatile approach to enhance device performance [41]. A typical SAM molecule is composed of a terminal group, a spacer, and an anchoring group, each differently contributing to the improvement of device performance. Properly selected terminal groups alter physical and chemical surface characteristics, as well as the dipole moment, which adjusts the work function of a substrate [42]. Anchoring group contributes to the bonding strength of SAM molecule to the surface of transparent conductive oxide (TCO), whereas spacer serves as a link between terminal and anchoring groups, creating an efficiently arranged molecular packing [43, 44]. A unique ability to self‐organize into well‐ordered structures at the molecular level enables precise control over the interfacial properties, serving as effective passivating layers, mitigating defects in the perovskite film, and facilitating better electron or hole extraction [45, 46, 47, 48]. Additionally, newly developed SAMs help to minimize the typical quantity of material used as HTL, prevent doping procedures, ensure the durability of devices, and show great potential for a large‐area PSC manufacturing owing to facile deposition techniques [49, 50, 51]. Even though these molecules have many previously mentioned positive properties, the effect of different anchoring groups to surface wettability, energy level alignment, and overall photovoltaic performance needs to be further investigated.

In this work, a novel approach to combine two promising ideas of enamine chemistry and self‐assembling technique was used to engineer a series of enamine‐based SAMs featuring various anchoring groups such as ─COOH, ─PO_3_H_2_, ─CH_2_─COOH, ─CH_2_─PO_3_H_2_, ─CH═C(CN)─COOH, and ─CH═C(CN)─PO_3_H_2_. Specifically selected anchors were chosen for their known advantages, such as reduction of nonradiative recombination, improvement of perovskite crystallinity, effective perovskite passivation and charge delocalization, thereby enhancing the overall PCE of PSC devices [52, 53, 54, 55, 56]. It was discovered that enamine‐based SAMs help to demonstrate highly promising device efficiency values surpassing 25%. The champion mini‐module realized a PCE of 23.14%. These findings show that meticulous alteration of enamine‐based SAMs' anchoring groups produces highly effective solar cells that have an excellent chance of developing into photovoltaic devices for mass production.

## 2 Results and Discussion

A schematic representation of enamine‐based SAMs with various anchoring groups such as carboxylic acid, acetic acid, cyanoacrylic acid, phosphonic acid, methylene phosphonic acid, cyanovinyl phosphonic acid (**CO1**, **CO2**, **CO3**, **PO1**, **PO2**, **PO3**, respectively) are shown in Figure 1a. Six novel SAMs were functionalized via simple enamine condensation by reaction of a primary amine with 2,2‐bis(4‐methoxyphenyl)acetaldehyde in the presence of (+/−)camphor sulfonic acid (CSA) [36]. The chemistry of enamines is a unique approach to conduct a target product under ambient conditions with only one byproduct—water. Cyano group containing intermediate compounds were synthesized via Knoevenagel condensation reaction. The cyanovinyl fragment was introduced as a multifunctional structural unit to extend molecular conjugation, enhance the push‐pull electronic character of the molecule, and potentially contribute to interfacial defect passivation through the cyano functionality. Detailed synthetic procedures and characterization of compounds (NMR spectroscopy, elemental analysis, mass spectrometry) are described in the Supporting Information (SI).

**FIGURE 1:**
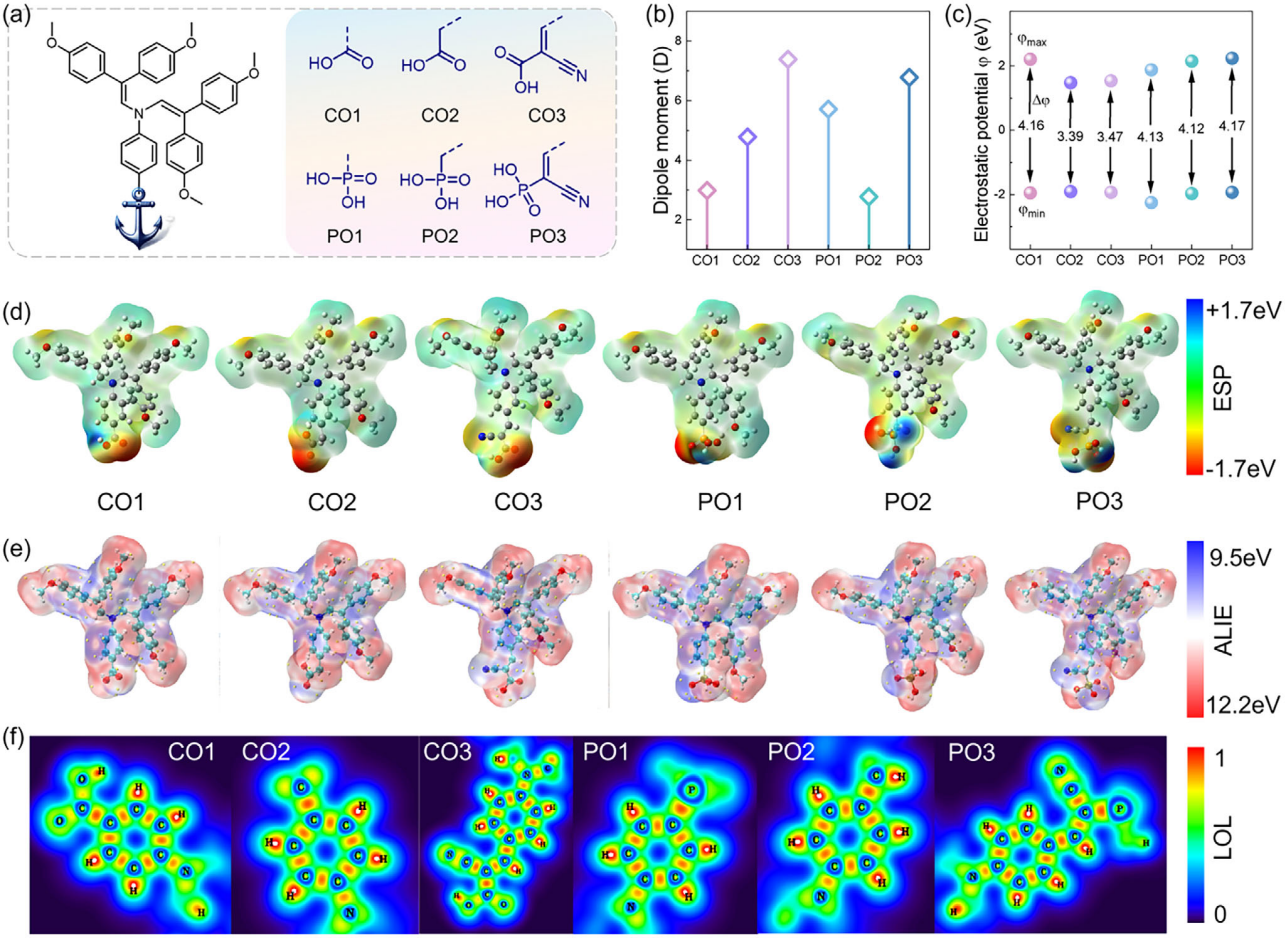
Molecular structures and quantum chemical properties of anchored enamine‐based SAMs. (a) Schematic representation of enamine‐based SAM chemical structures containing different anchors. (b) Dipole moment values. The unit is Debye. (c) Minima and maxima of electrostatic potential (ESP) on the surface. (d) ESP maps on van der Waals (vdW) surface (0.001 a.u. isosurface) of enamine‐based SAMs. (e) Average local ionization energy (ALIE) maps on vdW surface of enamine‐based SAMs. Yellow spheres indicate ALIE minima on this surface. (f) Color‐filled maps of localized orbital locator (LOL) of anchoring groups on benzene ring in enamine‐based SAMs. The atoms noncoplanar with benzene are not shown.

Originally, we investigate the structural characteristics of enamine‐based SAMs and elucidate the design principle by quantum chemical calculation and wave function analysis by utilizing density functional theory (DFT). (Computational details can be found in the SI) [57, 58, 59, 60] Electrostatic potential (ESP) measures interaction energy between a unit charge at a given point and the present system without charge transfer and charge polarization effects [58]. ESP on vdW surface is highly related to intermolecular electrostatic interactions. The ESP maps of enamine‐based SAMs are shown in Figure 1d and Figure S1. The ESP is slightly negative in the area surrounding methoxy groups due to high electronegativity of oxygen atoms, which leads to interactions with under‐coordinated Pb^2+^ at the buried interface of perovskite. Besides, the negative charge is mainly focused on oxygen atoms in anchoring groups. It is worth mentioning that the cyano groups in **CO3** and **PO3** are also negatively charged, indicating the electron‐withdrawing nature of cyano group. The positive charge is mainly distributed around hydrogen atoms. Besides, **CO3** and **PO3** exhibit the dipole moment of 7.39 D and 6.78 D, respectively (Figure 1b), which are larger than that of Me‐4PACz and 2PACz (3.40 D and 2.82 D, respectively). Moreover, we performed quantitative molecular surface analyses to calculate ESP maxima and minima on the vdW surface of enamine‐based SAMs. In Figure 1c, the differences between ESP minima and maxima are around 3–5 eV, which suggests obvious positive and negative charge separation, and thus contributing to hole transfer.

The alkyl linker‐based SAMs have been widely used in PSCs such as Me‐4PACz and 2PACz (Figure S2), yet they exhibit limited hole transfer capability due to the insulating alkyl linkers as well as poor stability due to the isolated and nonconjugated electron‐rich moiety [50]. While the conjugated enamine core links the two methoxy diarylamines and the anchoring group, enabling extended electron delocalization over the large aromatic system, which benefits in hole extraction and distinctive molecular orbital distribution. As shown in Figure S3, the overlapped highest occupied molecular orbitals (HOMO) and lowest unoccupied molecular orbitals (LUMO) of enamine‐based SAMs could enhance the UV stability of SAM films and PSCs [50, 61].

Next, we employ the average local ionization energy (ALIE) to study enamine‐based SAMs. The ALIE is a real space function, which reflects ionization energy of an electron in a local region. Generally, a lower ALIE indicates the electrons are bound weaker at this position, contributing to higher reactivity of the electrons. According to the ALIE maps in Figure 1e, active electrons are mainly distributed around benzene rings in enamine‐based SAMs (blue area), suggesting these fragments are relatively efficient to extract holes and facilitate hole transfer. For quantitative comparison, the minimal values of ALIE on the vdW surface of some other molecules containing π electrons are given in Figure S4. The corresponding values of the enamine‐based SAMs (varying from 8.08 to 8.64 eV) are close to those of 9*H*‐carbazole (8.56 eV) and Me‐4PACz (8.52 eV) but lower than benzene (9.17 eV) and V1036 (8.71 eV). In addition, the fragments of enamine core with methoxy diarylamines (denoted as E1 and E2 in Figure S4) are investigated, and their minimal values of ALIE are very low (8.17 eV and 7.60 eV for E1 and E2, respectively), confirming the strong electron‐donating ability of enamine linked methoxy diarylamines. Moreover, Fukui function is a real space function, which reveals the reactive sites for nucleophilic, electrophilic, or radical attack. As shown in Figure S5, Fukui function *f^−^* (the purple isosurfaces) of enamine‐based SAMs spreads over the whole molecule, indicating the conjugated enamine‐based SAMs are favorable for an electrophilic attack. Nevertheless, Fukui function *f* ^−^ mainly distributes on the carbazole ring in 2PACz and Me‐4PACz rather than the whole molecule. The enamine‐based SAMs are prone to donate active electrons (i.e., receive holes), and thus facilitate hole extraction and transport.

Furthermore, we employ various widely adopted and distinctive visualization methods to investigate the noncovalent interactions (NCI) of enamine‐based SAMs. Firstly, the interaction region indicator (IRI) analysis is introduced for visualization of covalent and noncovalent interactions in real space. The colored isosurfaces in the IRI map of enamine‐based SAMs in Figure S6 clearly reveal various interactions, such as polar and nonpolar covalent bonds (blue area), steric hindrance (red area), and vdW interactions (green area). The distinctive enamine linked methoxy diarylamines lead to *π*‐*π* interactions between adjacent benzene rings (the green isosurfaces in Figure S6), implying the intramolecular electron delocalization. For comparison, only limited *π*‐*π* interactions with small areas are observed in V1036 due to its lack of enamine core linker (Figure S7). Meanwhile, the enamine linked methoxy diarylamines not only enhance intramolecular *π*‐*π* interactions (Figure S7) but also distort the molecular skeleton due to steric hindrance and thus preventing SAM aggregation.

The localized orbital locator (LOL) and the electron localization function (ELF) are both dimensionless functions with a range of [0,1] for visual analysis of covalent interactions. In the color‐filled maps of ELF and LOL of anchoring groups on benzene ring, **CO3** and **PO3** demonstrate superior coplanarity of the fragments due to the electron‐withdrawing cyanovinyl group (Figure 1f; Figure S8), which benefits ordered molecular stacking and optimized interfacial contact with perovskite. To sum up, based on molecular engineering, the methoxybenzene head, enamine core linker, and multiple anchors are designed and elucidated by quantum chemical calculation and wave function analysis. We reveal that introducing the cyanovinyl group to enamine‐based SAMs increases the potential for hole transfer in PSCs.

To gain molecular‐level insight into the organization of SAMs on indium oxide, we performed molecular dynamics (MD) simulations on two representative systems: the conventional 2PACz SAM and our newly designed **PO3** SAM (MD details in SI). Both molecules anchor to the substrate via phosphonate groups but differ in their headgroup structures: 2PACz contains a planar carbazole unit, whereas **PO3** has a bulky 3D headgroup ((2‐(4‐(bis(2,2‐bis(4‐methoxyphenyl)vinyl)amino)phenyl)) and features a nitrile (–CN) group adjacent to the phosphonate anchor (Figure S9a). To estimate the SAM coverage on ITO, we approximated the volume of each SAM molecule, calculated the minimum area it occupies on the substrate, and arranged the molecules accordingly. We also performed preliminary energy minimizations by placing a few SAM molecules at varying distances from the substrate to observe how they adsorb and identify preferred binding orientations. The final binding configuration was determined based on these observations, supported by DFT‐level results on phosphonate anchors reported in the literature [62, 63, 64].

During the simulations of **PO3** and 2PACz SAMs, we observed two distinct behaviors (Figure 2; Movies S1–S5). The **PO3** SAM forms a uniform, more upright, and well‐dispersed monolayer, with molecules exhibiting limited but natural orientational freedom (Figures 2a and 3a–c; Movies S3–S5). In contrast, 2PACz molecules aggregate into clusters and form isolated islands (Figures 2b and 3d; Movies S1 and S2). As the simulation movies illustrate, the planar carbazole moiety in 2PACz readily seeks neighboring carbazole units to establish *π*–*π* interactions, driving lateral aggregation. This aggregation is verified by the green isosurfaces in IGMH maps of 2PACz dimer and trimer (Figure 3e; Figure S9b). In contrast, the pronounced steric hindrance of the bulky **PO3** headgroups prevents any strong intermolecular association, allowing the monolayer to remain uniformly dispersed throughout the simulation. Alongside steric effects, the dipole moment also supports the more upright (less tilted) and uniformly distributed arrangement of **PO3** monomers. The nitrile group adjacent to the phosphonate anchor withdraws electron density, generating a strong vertical dipole that energetically favors an upright orientation and minimizes lateral dipole–dipole repulsion.

**FIGURE 2:**
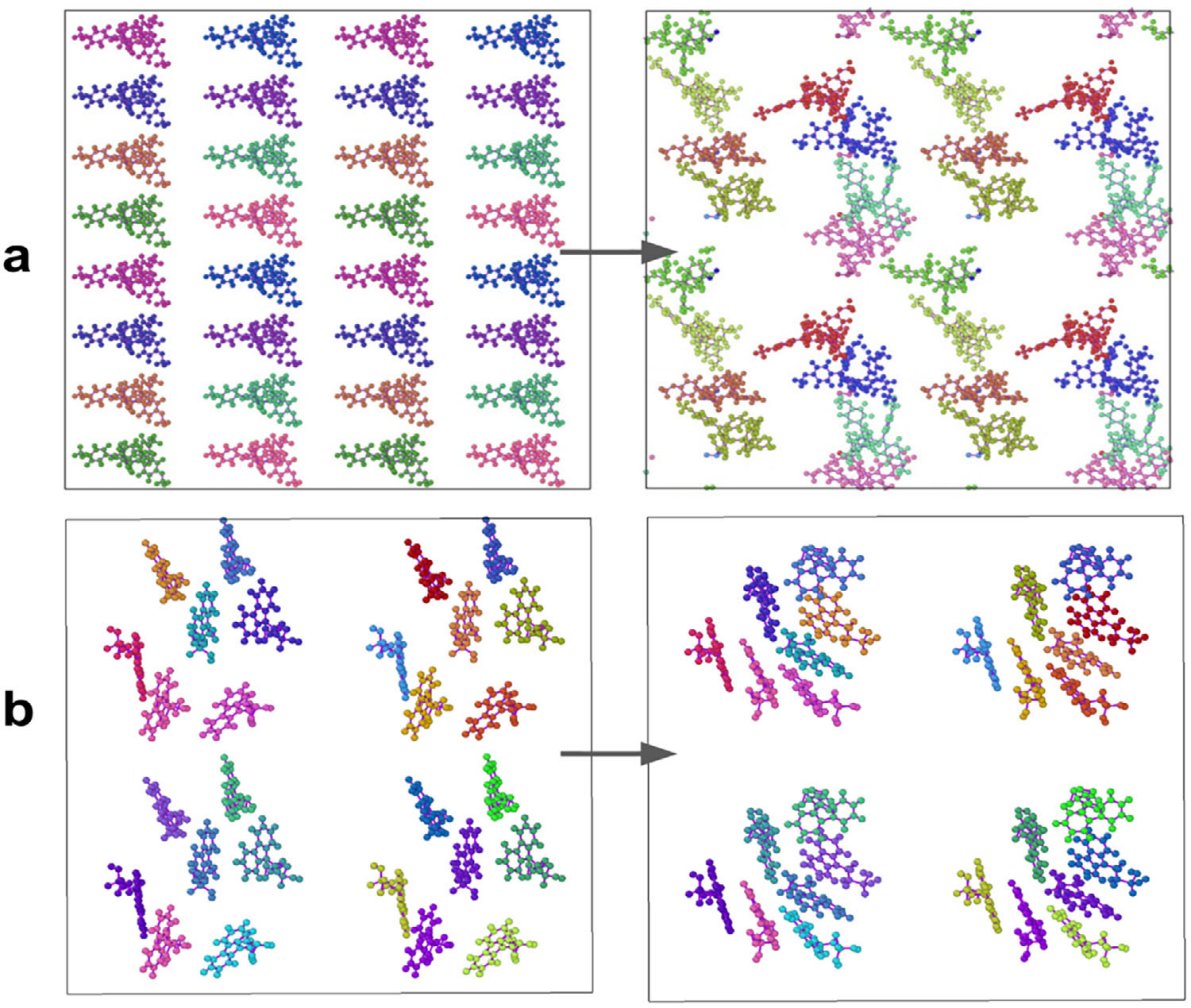
Top view of the distribution of SAM molecules before and after MD simulation (the substrate is removed intentionally from the pictures for clarity). (a) Top view of **PO3** molecules before (left) and after (right) equilibration. (b) Top view of 2PACz molecules before (left) and after (right) equilibration.

**FIGURE 3:**
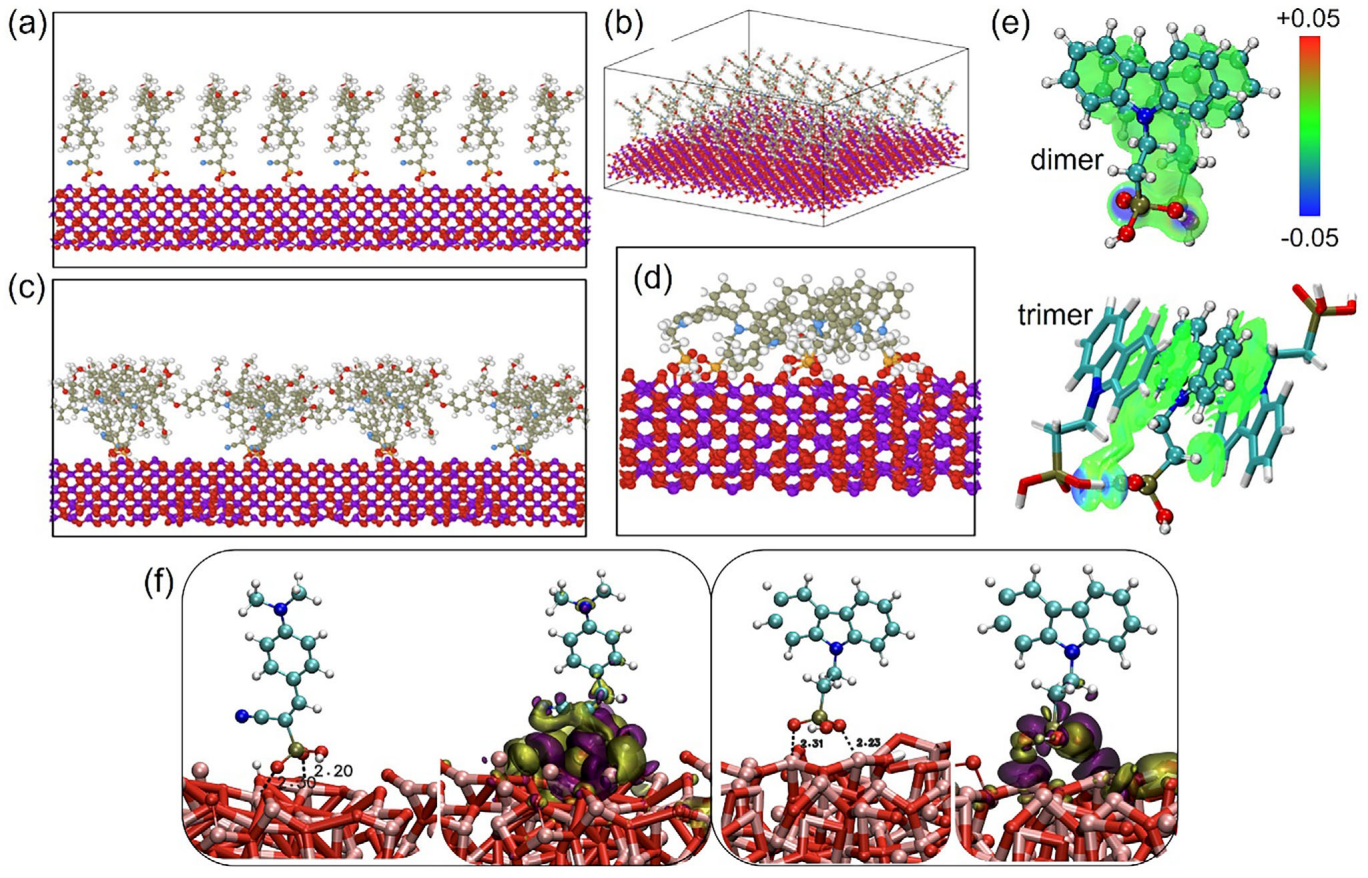
Side view of the distribution of SAM molecules before and after MD simulation on the indium oxide substrate. (a,b) Arrangement of maximum coverage for **PO3** molecules before equilibration. (c) Arrangement of maximum coverage for **PO3** molecules after equilibration. (d) Side view of 2PACz molecules aggregating into clusters. (e) Independent gradient model based on Hirshfeld partition (IGMH) maps of 2PACz dimer and trimer with isovalue of 0.001 a.u. (f) The DFT‐optimized singly deprotonated bidentate binding mode of **PO3** (left block) and 2PACz (right block) on ITO: Optimized distances and charge density difference: electron accumulation (yellow) and electron depletion (purple), Isosurface set at 土0.0006.

We further performed slab‐based DFT calculations of SAMs on ITO slab to investigate the interaction between the anchoring group and the oxide surface. (Figure 3f). The ITO substrate was modeled using the In_2_O_3_(111) surface. As discussed in the previous section, the steric hindrance introduced by the methoxybenzene groups suppresses excessive aggregation, while the *π*–*π* interactions between the aromatic head groups govern the packing and cluster arrangement of the SAMs. Since these structural effects have already been characterized, the slab calculations were designed specifically to investigate the interfacial chemistry between the anchoring group and the oxide surface. To this end, the electronically nearly inert methoxybenzene head groups were omitted, and only the linker and phosphonic acid anchoring group were retained. This truncated model preserves the electronic interactions governing adsorption, charge redistribution, and work‐function modification at the oxide interface, while substantially reducing the computational cost of the slab calculations. Based on the results presented in this work, the **PO3** linker was selected as the representative engineered SAM and compared with the widely used 2PACz molecule. For both systems, the initial adsorption geometry was constructed using a bidentate phosphonic acid binding configuration, which has been identified as the predominant adsorption mode of phosphonic acid SAMs on ITO surfaces in previous XPS/UPS studies [66]. Anticipating our results, the calculated adsorption energy indicates strong thermodynamically favorable binding of the phosphonic acid linker to the In_2_O_3_(111) surface (Figure 4).

**FIGURE 4:**
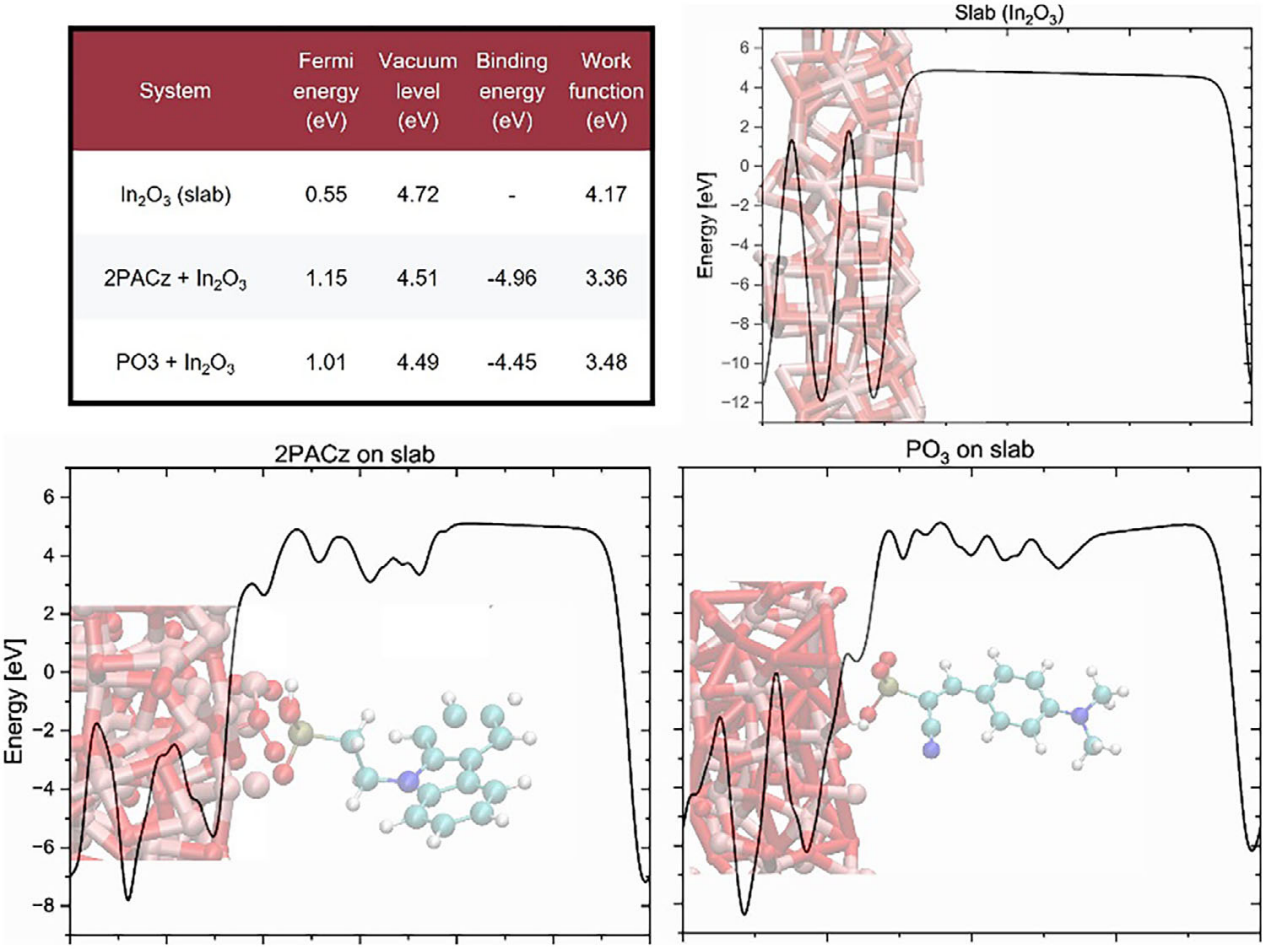
Table of calculated binding energies and work functions together with electrostatic potential profile of the ITO slab modified by 2PACz and **PO3**, SAM molecules (energy along the cell height).

The calculated electrostatic potential profiles of the clean and SAM‐functionalized In_2_O_3_(111) surfaces (Figure 4) demonstrate that adsorption of the SAM modifies the surface potential and results in an increase in the work function. Compared with the widely used 2PACz, the **PO3** linker induces a slightly larger work‐function increase, leading to a more favorable energy‐level alignment for hole extraction. Charge‐density difference analysis further reveals pronounced electron accumulation around the phosphonate anchoring group and corresponding electron depletion near the surface, evidencing interfacial charge redistribution and the formation of an interface dipole. This behavior is attributed to the electron‐withdrawing nature of the engineered linker, which shifts the electronic structure toward more favorable hole‐selective characteristics.

To investigate the thermal stability of synthesized compounds, thermogravimetric analysis (TGA) measurements were conducted, and the results are demonstrated in Figure 5 and summarized in Table 1. The thermal decomposition temperatures (*T*_dec_) that correspond to 5% weight loss of **CO1**, **CO2**, **CO3**, **PO1**, **PO2,** and **PO3** were estimated to be 361, 319, 272, 216, 251, and 262°C, respectively, which indicate that all synthesized SAMs have adequate thermal stability for conventional device operation. Interestingly, two HTLs with carboxyl group **CO1** and **CO2** showed a rapid weight loss, indicating that they undergo sublimation instead of thermal decomposition. Therefore, monolayers of such compounds could be formed using vacuum decomposition methods, thus expanding their applicability.

**FIGURE 5:**
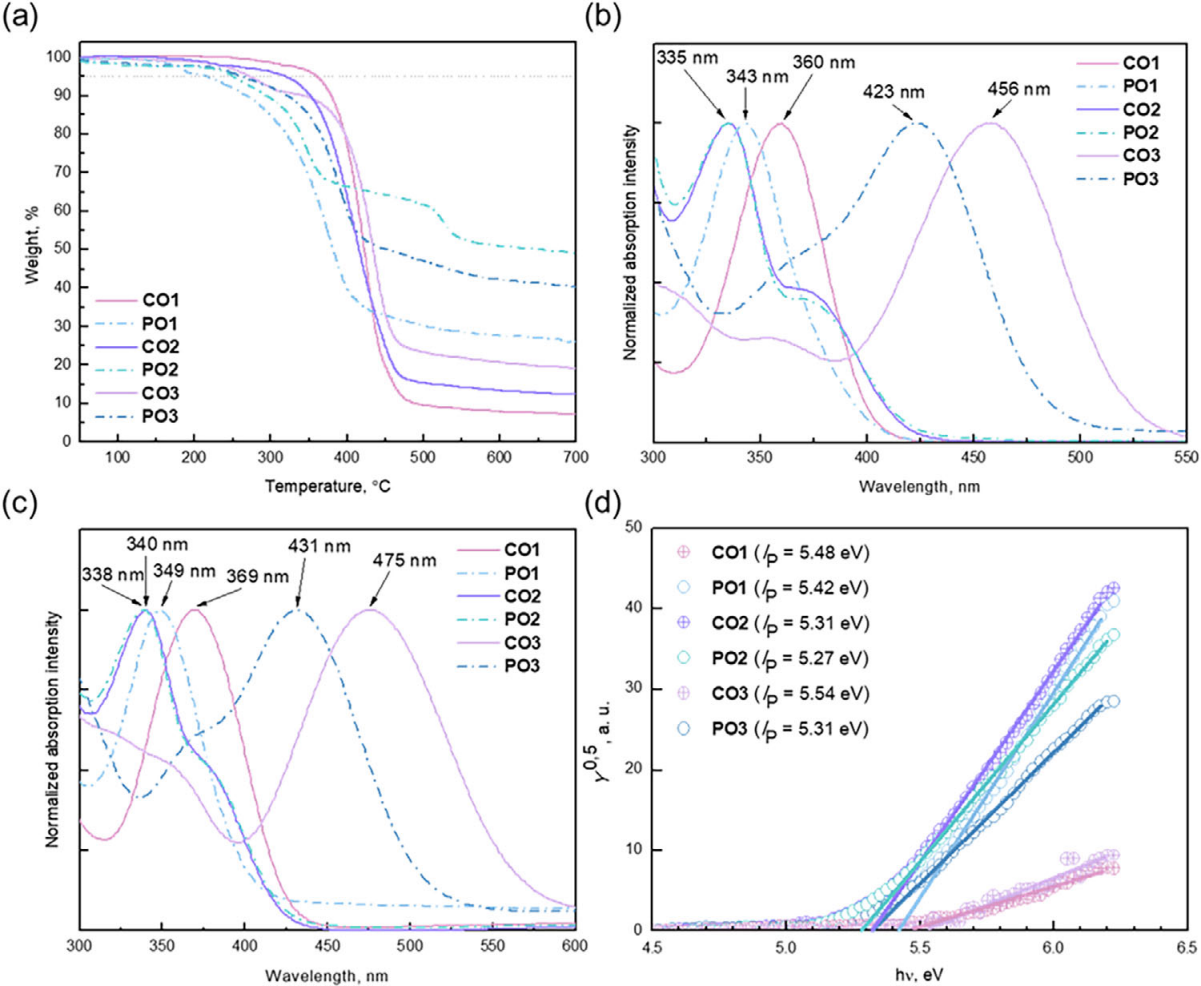
Thermal, optical and photophysical properties of anchored enamine‐based SAMs. (a) Thermogravimetric analysis (TGA) data (heating rate of 10°C min^−1^, N_2_ atmosphere). (b) Absorption spectra in THF solutions (10^−4^ M). (c) Absorption spectra of HTMs thin films. (d) PESA spectra.

**TABLE 1:** Thermal, optical and photophysical properties of enamine‐based SAMs.

ID	*T*_dec_ (°C)^a^	*λ*_max_ (nm)^b^	*λ*_max_ (nm)^c^	*I*_P_ (eV)^d^
**CO1**	361	261; 360	287; 369	5.48
**PO1**	216	262; 343	287; 349	5.42
**CO2**	319	263; 335	289; 340	5.31
**PO2**	251	263; 335	289; 338	5.27
**CO3**	272	257; 456	287; 475	5.54
**PO3**	262	267; 423	287; 431	5.31

^a^ Decomposition (*T*_dec_) temperatures observed from TGA, respectively (10°C min^−1^, N_2_ atmosphere).

^b^ Absorption spectra were measured in THF solutions (10^−4^ m).

^c^ UV–vis absorption spectra of HTMs thin films.

^d^ Ionization potentials of the films measured using PESA.

To evaluate optical properties, ultraviolet–visible (UV–vis) absorption spectra were measured in THF solutions and from thin films. The spectra are shown in Figure 5b,c, the related data are summarized in Table 1. The change of the anchoring group significantly influences the absorption. The absorption maximum (*λ*_max_) of SAMs containing carboxyl and phosphonic acid groups, **CO1** and **PO1**, respectively, was observed at 360 and 343 nm. The addition of methylene group in **CO2** and **PO2** shifts absorption maximum hypsochromically for both compounds (*λ*_max_ = 335 nm), having more noticeable effect of 25 nm on the HTM with carboxyl group. As predicted, compounds with incorporated powerful electron‐withdrawing cyano groups (**CO3**, **PO3**) showed conspicuous bathochromic shifts. Despite the visible‐light absorption of discussed molecules, the ultrathin nature of self‐assembled monolayer limits parasitic absorption losses, allowing efficient light harvesting by the perovskite active layer. The absorption spectra of SAMs in solution demonstrate comparative results with the UV‐vis spectra from thin films (Figure 5c).

To determine the HOMO energy levels of synthesized HTMs, the solid‐state ionization potential (*I*_P_) was measured using the photoelectron spectroscopy in air (PESA) method, the results are shown in Figure 5d and summarized in Table 1. The HOMO energy levels of **CO1**, **PO1**, **CO2**, **PO2**, **CO3**, and **PO3** were determined to be −5.48, −5.42, −5.31, −5.27, −5.54, and −5.31 eV, respectively. Enamine derivatives containing carboxyl anchoring group (**CO1**, **CO2**, and **CO3**) exhibit deeper HOMO values compared to the corresponding enamines containing phosphonic acid anchor (**PO1**, **PO2**, and **PO3**). The addition of methylene group (**CO2** and **PO2**) lowers the *I*_P_ values of HTMs by ≈0.15 eV. Overall, HOMO energy levels of the novel SAMs are suitable for the application in the PSCs.

Interfacial contact and charge transfer between SAMs and perovskite are vital to the performance of PSCs. Atomic force microscopy (AFM) and Kelvin probe force microscopy (KPFM) were conducted to investigate the surface uniformity and potential of SAMs films. As illustrated in Figure 6a, both **CO3** and **PO3** on ITO show uniform film morphology. The uniform and smooth surface optimizes the dense interfacial contact with perovskite layer. The contact potential distribution (CPD) maps of **CO3** and **PO3** are shown in Figure 6b and Figures S10 and S11. Compared to the average CPD value of **CO3** (‐0.165 V), the less negative CPD value of **PO3** (−0.124 V) with relatively uniform distribution indicates more p‐type characteristics of the buried interface properties, contributing to better hole conduction from perovskite to ITO/SAM [65].

**FIGURE 6:**
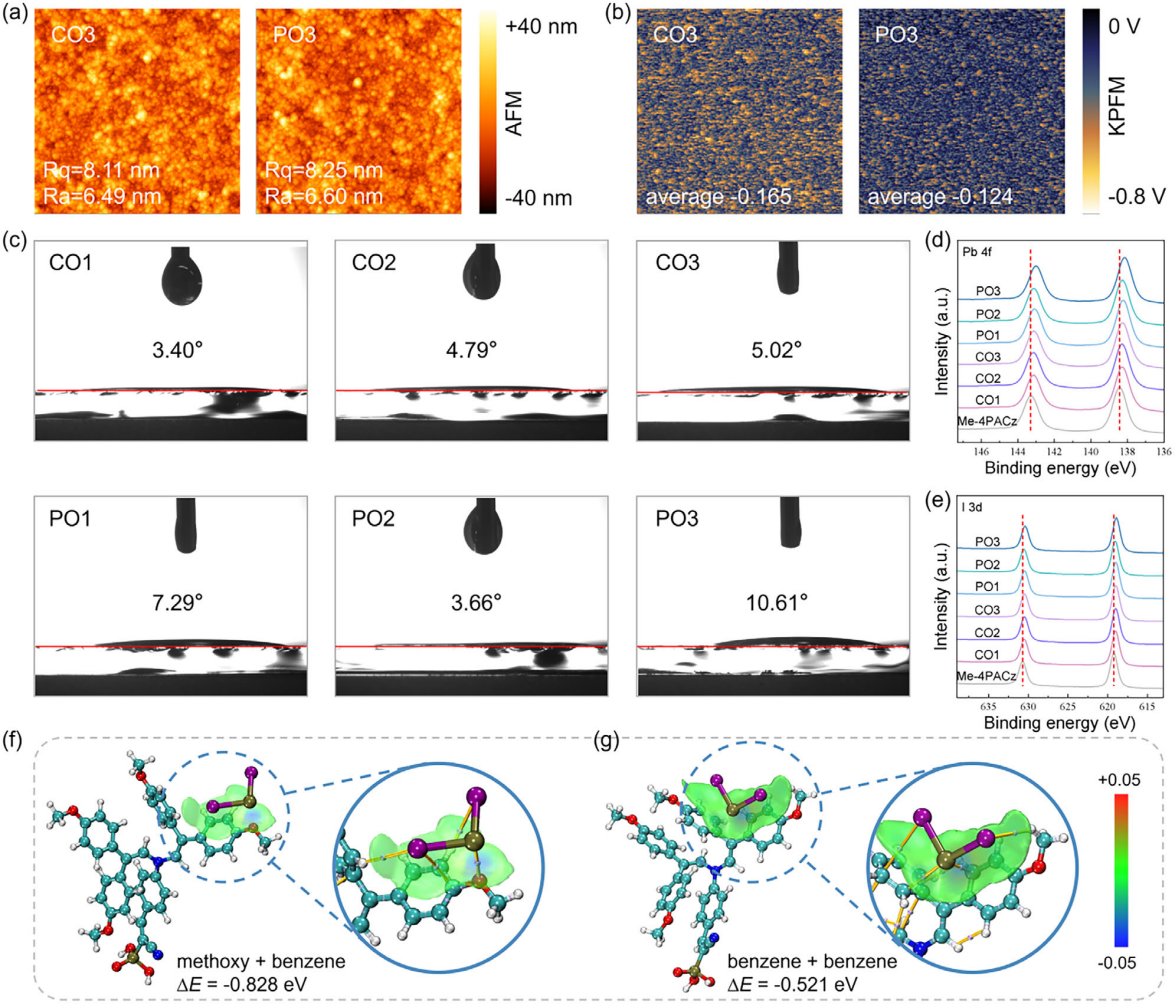
Interfacial contact and charge transfer between SAMs and perovskite. (a) AFM and (b) KPFM images of ITO/**CO3** and ITO/**PO3**. (c) Contact angles of differently anchored enamine‐based SAMs on ITO. The dropped solution is DMF and DMSO (4:1 in volume). (d) XPS Pb 4f spectra of perovskite films on ITO/SAM. (e) XPS I 3d spectra of perovskite films on ITO/SAM. (f,g) IGMH maps and topology analyses on electron density based on Atoms in Molecules (AIM) theory for **PO3** binding with PbI_2_. (f) **PO3** binds with PbI_2_ via methoxy + benzene. (g) **PO3** binds with PbI_2_ via benzene + benzene. The isosurface of IGMH is 0.001 a.u. Yellow lines are bond paths, and purple spheres on yellow lines correspond to the (3,‐1) critical points (i.e., bond critical point, BCP).

To indicate monolayer formation and evaluate surface wettability of differently anchored SAM films, contact angle (*θ*) measurements of differently anchored enamine‐based SAMs on ITO were performed. Firstly, we applied DMF and DMSO (4:1 in volume) as the dropped solution to study how the wettability of the buried layer influences perovskite crystallization. Presented by Figure 6c, all the differently anchored enamine‐based SAMs demonstrate relatively low contact angles, indicating the homogeneous distribution of SAM molecules without aggregation due to distorted molecular skeleton and the enamine linked methoxy diarylamines. The methoxy groups increase the wettability of perovskite precursor on SAM films. Interestingly, **CO3** and **PO3** show slightly larger contact angles of 5.02° and 10.61°, probably originating from the steric hindrance of cyanovinyl acid groups. The moderate wettability to perovskite precursor leads to furtherance of compact interface of SAM layer and perovskite with less defects.

Furthermore, the water contact angles were measured, and results are presented in Figure S12. A significant change in *θ* is noticeable when comparing pristine ITO to ITO covered with SAM, indicating surface modification. Another obvious difference of contact angles is observed between SAMs containing carboxyl anchoring group (**CO1**, **CO2**, and **CO3**) and SAMs with phosphonic acid anchor (**PO1**, **PO2** and **PO3**), the first ones display clearly more hydrophilic surfaces with *θ* of 49, 46, and 42°, respectively, similar to the one of MeO‐2PACz (54°), than the second ones with *θ* of 78, 69, and 70°, respectively. A supplemental methylene group reduces contact angles of water droplets (49° to 46°, 78° to 69°), whereas the incorporation of cyano group has a different effect on both groups of compounds: slightly improves wettability for carboxyl anchors containing SAMs (46° to 42°) and does not exhibit significant change of *θ* for compounds with phosphonic acid anchoring group (69° to 70°). Overall, SAMs with incorporated carboxyl anchoring groups exhibit clear trends of lower water contact angles, indicating improved wettability and surface coverage.

The chemical environment of the perovskite on enamine‐based SAMs was studied by XPS measurements (Figure S13). In comparison with perovskite on Me‐4PACz, Pb 4f 5/2 and Pb 4f 7/2 peaks of the perovskite on enamine‐based SAMs shift toward lower binding energies due to reduced undercoordinated Pb^2+^ (Figure 6d). Specifically, the methoxy groups in enamine‐based SAMs are negatively charged (Figure 1d), favoring the Lewis acid‐base interactions with undercoordinated Pb^2+^ to suppress interfacial defects. In Figure 6e, the I 3d spectra of the perovskite on enamine‐based SAMs shift toward lower binding energies because of the chemical environment change of Pb^2+^. Visualized by IGMH maps and topology analyses based on the atoms‐in‐molecules (AIM) theory (Figure 6f,g; Figure S14), **PO3** binds with PbI_2_ via methoxy‐benzene or two benzene rings. The IGMH isosurface (green area), bond paths (yellow lines), and bond critical points (purple spheres) jointly confirm the interaction area. While the former (Figure 6f, ∆*E* = −0.828 eV) exhibits stronger interaction than the latter (Figure 6g, ∆*E* = −0.521 eV), indicating the Lewis acid‐base interactions between the methoxy groups and undercoordinated Pb^2+^. When binding with PbI_2_ via methoxy‐benzene, **PO3** shows stronger interaction (∆*E* = −0.828 eV) than that of Me‐4PACz (∆*E* = −0.703 eV), since methoxy is better electron‐donor than carbazole or methyl.

Optimized interface between SAMs and perovskite is conducive to regulating perovskite crystallization. Top‐view scanning electron microscopy (SEM) was performed to investigate the perovskite crystalline grains at the top and buried interface. In Figure 7a and Figure S15, the perovskite films on **CO3** and **PO3** demonstrate the largest grains with minor PbI_2_ particles on the surface. Similarly, the buried interface of perovskite films based on **CO3** and **PO3** shows significantly improved morphology with fewer pinholes (Figure S16). X‐ray diffraction (XRD) patterns show that all the perovskite films tend to grow along (100) and (200) crystallographic orientations. Additionally, compared with Me‐4PACz, perovskite films on **CO3** and **PO3** show enhanced diffraction intensity peaks and reduced full‐width at half‐maximum (FWHM) of both the top and bottom surface (Figure 7b,c; Figure S17), indicating orderly grown perovskite with superior crystallinity, which benefits from dense packed enamine‐based SAM films and improved interfacial wettability.

**FIGURE 7:**
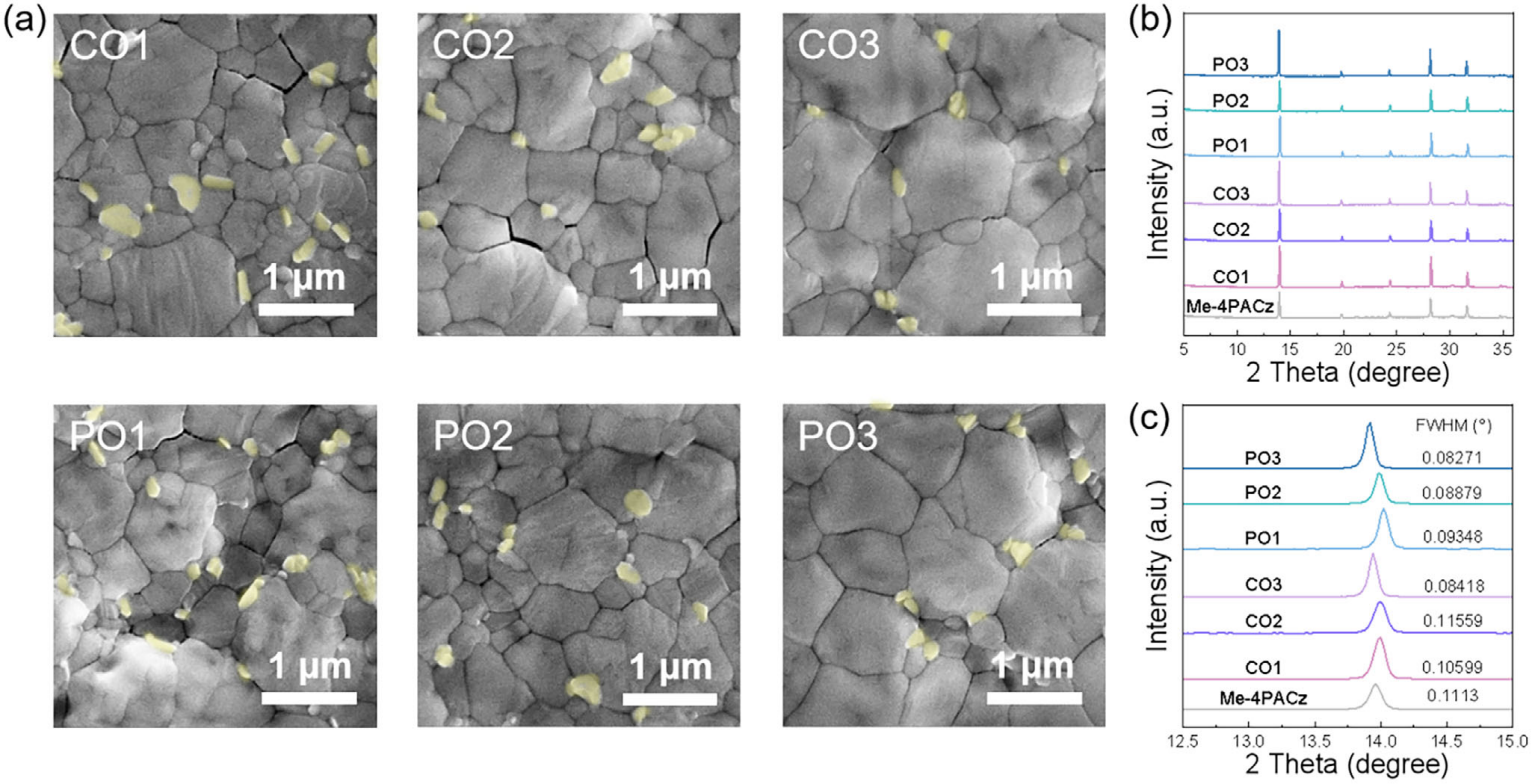
Optimized perovskite crystallization on anchored enamine‐based SAMs. (a) Top view SEM images of the perovskite films deposited on ITO/SAM. PbI_2_ particles are marked by yellow. (b) The XRD patterns of perovskite films deposited on ITO/SAM. (c) Enlarged XRD patterns of (100) peaks of perovskite.

We investigated the optoelectronic properties of perovskite films and devices. As shown in the steady‐state photoluminescence (PL) spectroscopy and time‐resolved photoluminescence spectroscopy (TRPL), perovskite films on **CO3** and **PO3** display the strongest PL intensity and prolonged TRPL lifetime (Figure 8a; Figure S18a), manifesting the suppressed non‐radiative recombination at the buried interface of perovskite. Based on PL mapping profiles (Figure 8b; Figure S19), compared with Me‐4PACz, the perovskite films on **CO3** and **PO3** show higher PL intensity, as well as narrower wavelength distribution and full width at half maximum (FWHM), suggesting the defect suppression and better interface quality of enamine‐based SAMs.

**FIGURE 8:**
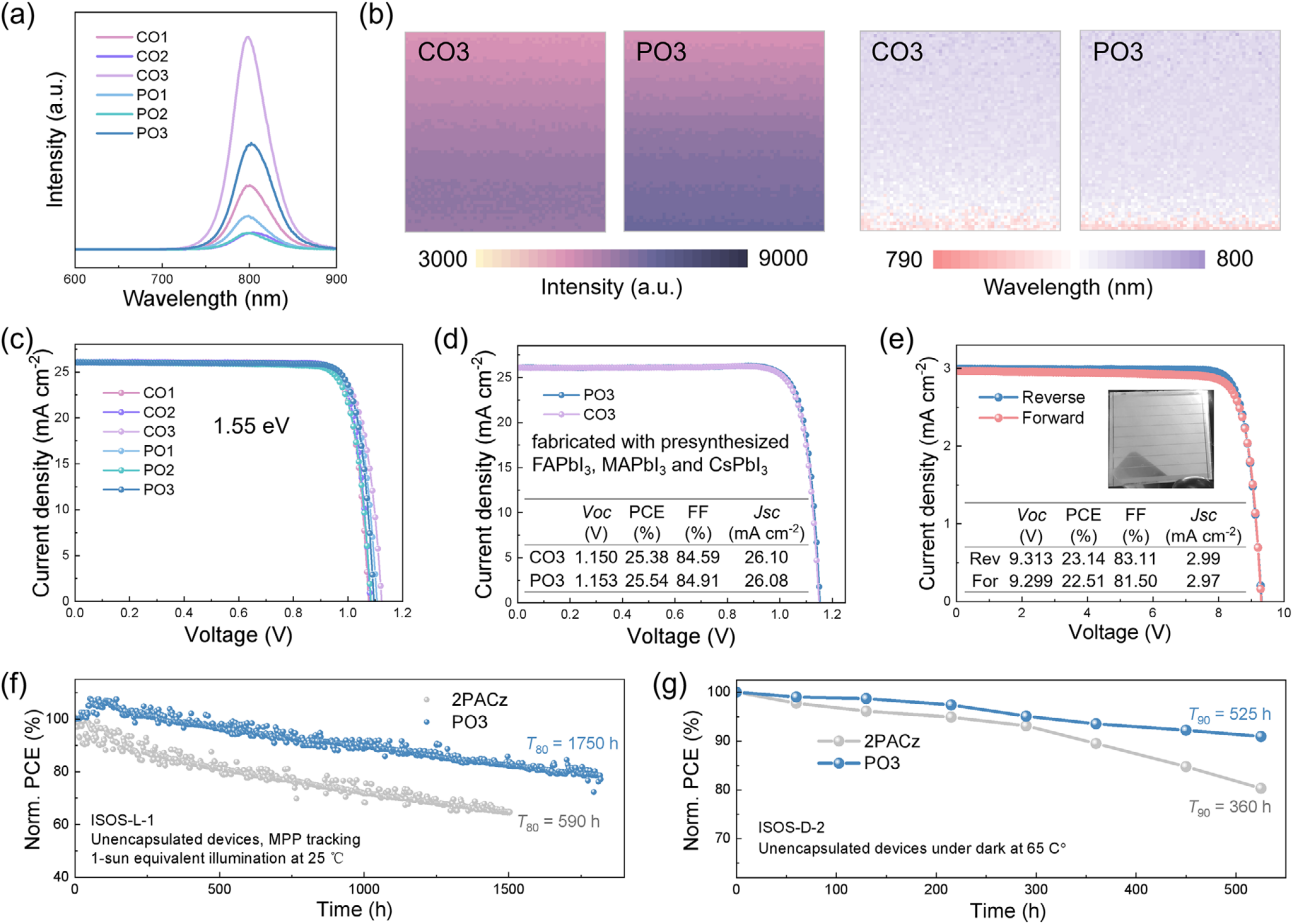
Defect management and device performance. (a) Steady‐state photoluminescence (PL) spectra of the perovskite films on ITO/SAM. (b) PL mapping images on a 40 µm × 40 µm region of perovskite films deposited on ITO/SAM, including PL intensity and wavelength. (c) *J–V* curves of the devices based on 1.55 eV perovskite (typical bandgap). (d) *J–V* characteristics of the champion devices fabricated with presynthesized FAPbI_3_, MAPbI_3_, and CsPbI_3_. (e) *J–V* characteristics and photo of the champion module based on **PO3** under reverse and forward scan. The active area (AC) and aperture area (AP) are 27 and 29.7 cm^2^, respectively. (f) ISOS‐L‐1 stability protocol under the maximum power point (MPP) tracking conditions under 1‐sun equivalent illumination at 25°C in nitrogen. (g) ISOS‐D‐2 stability protocol under dark storage in nitrogen at 65°C.

Based on electrochemical impedance spectroscopy (EIS), the device with **PO3** displays larger charge recombination resistance (*R*_rec_) than that with Me‐4PACz (Figure S18b), confirming weak trap‐assisted charge recombination and thus improved interfacial carrier transport. Compared with the device with Me‐4PACz, the device with **PO3** shows longer transient photovoltage (TPV) time decay and shorter transient photocurrent (TPC) time decay (Figure S18c,d), indicating suppressed carrier recombination and ultrafast charge separation, respectively.

The configuration of inverted PSCs is ITO/SAM/perovskite/C_60_/BCP/Ag, and PSCs were measured under AM 1.5G (100 mW cm^−2^) illumination. For 1.55 eV perovskite, performance comparison is shown in Figure 8c, Table 2 and Figure S1. Specifically, 1 mmol/L enamine‐based SAMs result in obvious improvements in comparison with Me‐4PACz (Figure S20). The champion device based on **PO3** realized a PCE of 24.24% with a *Voc* of 1.095 V, a *Jsc* of 26.05 mA cm^−2^ and a FF of 84.93% under reverse scan, while the champion device based on Me‐4PACz achieved a PCE of 21.98% with a *Voc* of 1.099 V, a *Jsc* of 25.95 mA cm^−2^ and a FF of 77.07% (Figure 8c; Figure S21). The *Jsc* values obtained from the *J–V* curves match well (within 5% deviation) with the integrated *Jsc* obtained from the external quantum efficiency (EQE) spectra in Figure S22. Besides, PSCs based on enamine‐based SAMs display reduced hysteresis index (HI) than those of Me‐4PACz (Figure S23). The extremely high FF of PSCs based on enamine‐based SAMs originates from reduced interface defects and optimized hole extraction. Furthermore, by employing presynthesized FAPbI_3_, MAPbI_3_ and CsPbI_3_ nanocrystals as solutes to fabricate devices, the best device based on **PO3** delivered a PCE of 25.54% with a *Voc* of 1.153 V, a *Jsc* of 26.08 mA cm^−2^ and a FF of 84.91% under reverse scan (Figure 8d). Moreover, 1.68 eV wide bandgap PSCs based on **PO3** realized a PCE of 22.36% with preferment of *Voc* and FF than that of 2PACz (Figure S24 and Table S2), indicating the universality of enamine‐based SAMs. To confirm the scalability of the mixed‐SAM strategy, we fabricated mini‐modules with an aperture area of 29.7 cm^2^. The champion module realized a PCE of 23.14%, with a *Voc* of 9.313 V, a *Jsc* of 2.99 mA cm^−2^, and an FF of 83.11% under reverse scan (Figure 8e).

**TABLE 2:** Photovoltaic parameters of 1.55 eV champion PSCs on enamine‐based SAMs under reverse scan.

	*Voc* (V)	PCE (%)	FF (%)	*Jsc *(mA cm^−2^)
**CO1**	1.076	23.77	84.78	26.06
**CO2**	1.082	23.98	84.94	26.10
**CO3**	1.124	24.07	82.34	26.02
**PO1**	1.106	23.99	83.17	26.07
**PO2**	1.084	23.50	83.22	26.05
**PO3**	1.095	24.24	84.93	26.05

According to the ISOS‐L‐1 stability protocol, the unencapsulated **PO3**‐based device retained 80% of the initial PCE after 1750 h at maximum power point (MPP) tracking under 1‐sun equivalent illumination at 25°C in nitrogen (Figure 8f). Under the ISOS‐D‐2 test, the unencapsulated **PO3**‐based device maintained 90% of its initial PCE for 525 h under dark at 65°C in nitrogen (Figure 8g). In summary, the robust stability of enamine‐based SAMs leads to an optimized interface, contributing to enduring photovoltaic operation.

## 3 Conclusions

We have developed a family of enamine‐based SAMs by the anchor engineering with a direct comparison between a set of six different structural anchoring units of hole‐selective self‐assembled monolayers. We successfully demonstrate the enhanced *π*‐electron delocalization and optimized interfacial contact via conjugated cyanovinyl phosphonic acid anchoring unit lead to the champion 1.55 eV PSC with a PCE of 25.54% and FF of 84.91% with improved stability. The best mini‐module displays a PCE of 23.14% with an FF of 83.11%. This work provides valuable insights for designing novel SAMs to advance molecular design towards efficient perovskite‐based photovoltaics.

## Author Contributions

**Yuxuan Yang**: conceptualization, data curation, investigation. **Deimante Krisiune**: methodology, investigation, writing – original draft. **Yongde Xu**: data curation, formal analysis, validation. **Kasparas Rakstys**: writing – review and editing, conceptualization, project administration, supervision. **Jianxing Xia**: software, validation, conceptualization. **Negar Ashari Astani**: software, writing – review and editing, project administration, supervision. **Chuanxiao Xiao**: data curation, investigation, methodology. **Ali Keshavarz Mohammadian**: software, data curation, formal analysis. **Hao Tian**: investigation, validation, formal analysis. **Yi Zhang**: writing – review and editing, project administration, supervision, resources. **Vytautas Getautis**: resources, supervision, project administration, funding acquisition. **Mahan Saberi Zafarghandi**: software, formal analysis, data curation.

## Conflicts of Interest

The authors declare no conflicts of interest.

## Supporting information




**Supporting File 1**: advs76950‐sup‐0001‐SuppMat.docx.

**Supporting File 2**: advs76950‐sup‐0002‐MovieS1.mp4.

**Supporting File 3**: advs76950‐sup‐0003‐MovieS2.mp4.

**Supporting File 4**: advs76950‐sup‐0004‐MovieS3.mp4.

**Supporting File 5**: advs76950‐sup‐0005‐MovieS4.mp4.

**Supporting File 6**: advs76950‐sup‐0006‐MovieS5.mp4.

## Data Availability

The data that support the findings of this study are available from the corresponding author upon reasonable request.
