# Supplementary material for: Anchor Engineering of Hole‐Selective Enamine‐Based Self‐Assembling Monolayers for Perovskite Solar Cells
Source: Adv Sci (Weinh). 2026 Aug 21;13(62):e76950. doi: 10.1002/advs.76950 (PMC13495742; doi:10.1002/advs.76950)
Supplement: Supplementary file 1 [file ADVS-13-e76950-s001.docx]

Supporting Information

**Anchor Engineering of Hole-selective Enamine-based Self-assembling Monolayers for Perovskite Solar Cells**

Deimante Krisiune^1^, Yuxuan Yang^2^, Yongde Xu^2^, Hao Tian^3^, Chuanxiao Xiao^3^, Jianxing Xia^2^, Mahan Saberi, Zafarghandi^4^, Ali Keshavarz Mohammadian^4^, Negar Ashari Astani^4,^*, Kasparas Rakstys^1,^*, Yi Zhang^2,^*, Vytautas Getautis^1,^*

^1^ Department of Organic Chemistry, Kaunas University of Technology, Kaunas 50254, Lithuania

^2^ Institute of Molecular Plus, School of Chemical Engineering and Technology, Tianjin University, Tianjin 300072, China

^3^ Ningbo Institute of Materials Technology and Engineering, Chinese Academy of Sciences, Ningbo 315201, China

^4^ Department of Physics and Energy Engineering, Amirkabir University of Technology, Iran

**GENERAL**

Chemicals required for the synthesis were purchased from Sigma-Aldrich Co., Ltd. (Sigma-Aldrich), Tokyo Chemical Industry Co., Ltd. (TCI) Europe and BLD Pharmatech, Ltd. (BLDpharm) and used as received without additional purification. 2,2-bis(4-methoxyphenyl)acetaldehyde was synthesized following the literature procedure^[1]^. ^1^H NMR spectra were recorded at 400 MHz on a Bruker Avance III spectrometer with a 5 mm double resonance broad band BBO z-gradient room temperature probe, ^13^C NMR spectra were collected using the same instrument at 101 MHz. The chemical shifts, expressed in ppm, were relative to tetramethylsilane (TMS). All the NMR experiments were performed at 25 °C. Reactions were monitored by thin-layer chromatography on ALUGRAM SIL G/UV254 plates and developed with UV light. Silica gel (grade 9385, 230–400 mesh, 60 Å, Aldrich) was used for column chromatography. Elemental analysis was performed with an Exeter Analytical CE-440 elemental analyzer, Model 440 C/H/N/. MS were recorded on Waters SQ Detector 2 Spectrometer using electrospray ionization (ESI) technique.

Thermogravimetric analysis (TGA) was performed on a Q50 thermogravimetric analyzer (TA Instruments) at a scan rate of 10 °C min^–1^ under nitrogen atmosphere. The values are given for a weight-loss of 5% (*T*_dec_).

UV–vis spectral analysis of the samples in solution (THF, 10^–4^ mol L^–1^) and in thin films was performed on a Perkin Elmer Lambda 35 UV–VIS spectrophotometer. The layer thickness of the solution is d = 1 mm. Diffraction grating crack width is 2 nm. Spectral recording speed is 2 nm s^-1^. The wavelength λ is given in nm.

The solid-state ionization potential (*I*_p_) of the layers of the synthesized compounds was measured by the electron photoemission in air method; ^[2,3]^ the measurement error is evaluated as 0.03 eV. The samples for the ionization potential measurement were prepared by dissolving materials in THF and were coated on Al plates pre-coated with ~0.5 μm thick methylmethacrylate and methacrylic acid copolymer adhesive layer. The thickness of the transporting material layer was 0.5-1 μm. More detailed description can be found in ^[4]^.

**DETAILED SYNTHETIC PROCEDURES**

**4-(bis(2,2-bis(4-methoxyphenyl)vinyl)amino)benzoic acid (CO1)**

4-Aminobenzoic acid (0.25 g, 1.82 mmol) was dissolved in toluene (8 mL + volume of the Dean-Stark trap), (+/-)camphor-10-sulphonic acid (0.42 g, 1.82 mmol) was added and the mixture was heated at reflux for 20 minutes. Afterwards, 2,2-bis(4-methoxyphenyl)acetaldehyde (1.17 g, 4.56 mmol) was added, and reflux continued using a Dean-Stark trap. After termination of the reaction (1.5 h, TLC, THF:*n*-hexane, 1:1) the obtained crystals were filtered off and washed with water and ethanol. The crude product was crystallized from THF and ethanol mixture (1:1, 80 ml). The obtained crystals were filtered off and washed with ethanol to collect **CO1** as a yellow solid (0.61 g, 55% yield). ^1^H NMR (400 MHz, DMSO-*d*_6_) δ: 12.54 (s, 1H); 7.88 (d, *J* = 8.4 Hz, 2H); 7.05 (d, *J* = 8.4 Hz, 2H); 6.93 (d, *J* = 8.5 Hz, 4H); 6.87 (d, *J* = 8.5 Hz, 4H); 6.66 (d, *J* = 8.3 Hz, 4H); 6.37 (d, *J* = 8.4 Hz, 4H); 5.74 (s, 2H); 3.81 (s, 6H); 3.69 (s, 6H) ppm. ^13^C NMR (101 MHz, DMSO-*d*_6_) δ: 167.13; 158.95; 158.67; 149.13; 133.18; 131.87; 131.56; 131.10; 130.07; 128.49; 125.59; 122.77; 115.15; 114.10; 113.14; 67.02; 55.31; 55.06; 25.13 ppm. Anal. calcd for C_39_H_35_NO_6_: C 76.33; H 5.75; N 2.28; found: C 76.65; H 6.01; N 2.38. C_39_H_35_NO_6_ [M^+^] exact mass = 613.25, MS (ESI) = 613.16.

**2-(4-(bis(2,2-bis(4-methoxyphenyl)vinyl)amino)phenyl)acetic acid (CO2)**

4-Aminophenylacetic acid (0.30 g, 1.99 mmol) was dissolved in toluene (12 mL + volume of the Dean-Stark trap), (+/-)camphor-10-sulphonic acid (0.46 g, 1.99 mmol) was added and the mixture was heated at reflux for 20 minutes. Afterwards, 2,2-bis(4-methoxyphenyl)acetaldehyde (1.07 g, 4.17 mmol) was added, and reflux continued using a Dean-Stark trap. After termination of the reaction (3.5 h, TLC, MeOH:DCM, 1:24) the reaction mixture was extracted with ethyl acetate. The organic layer was dried over anhydrous Na_2_SO_4_, filtered and solvent evaporated. The crude product was purified by column chromatography using acetone:*n*-hexane (v:v; 8:17) eluent. The obtained product was precipitated from acetone into 15-fold excess of *n*-hexane. The precipitate was filtered off and washed with hexane to collect **CO2** as a yellow solid (0.30 g, 24% yield). ^1^H NMR (400 MHz, DMSO-*d*_6_) δ: 12.30 (s, 1H); 7.20 (d, *J* = 8.0 Hz, 2H); 6.96 (d, *J* = 7.2 Hz, 2H); 6.93 – 6.82 (m, 8H); 6.64 (d, *J* = 9.1 Hz, 4H); 6.36 (d, *J* = 8.3 Hz, 4H); 5.66 (s, 2H); 3.80 (s, 6H); 3.69 (s, 6H); 3.52 (s, 2H) ppm. ^13^C NMR (101 MHz, DMSO-*d*_6_) δ: 173.05; 158.77; 158.43; 144.26; 133.61; 131.93; 130.38; 130.22; 130.10; 128.32; 128.08; 126.20; 116.11; 114.02; 113.09; 55.29; 55.05; 30.72 ppm. Anal. calcd for C_40_H_37_NO_6_: C 76.54; H 5.94; N 2.23; found: C 76.88; H 6.35; N 2.39. C_40_H_37_NO_6_ [M^+^] exact mass = 627.26, MS (ESI) = 626.70.

**(*E*)-3-(4-(bis(2,2-bis(4-methoxyphenyl)vinyl)amino)phenyl)-2-cyanoacrylic acid (CO3)**

3-(4-aminophenyl)-2-cyano-2-propenoic acid, synthesized as reported previously^[5]^, (0.30 g, 1.59 mmol) was dissolved in toluene (9.5 mL + volume of the Dean-Stark trap), (+/-)camphor-10-sulphonic acid (0.37 g, 1.59 mmol) was added and the mixture was heated at reflux for 20 minutes. Afterwards, 2,2-bis(4-methoxyphenyl)acetaldehyde (0.86 g, 3.35 mmol) was added, and reflux continued using a Dean-Stark trap. After termination of the reaction (3.5 h, TLC, MeOH:TEA:DCM, 1:1:23) the reaction mixture was extracted with ethyl acetate. The organic layer was dried over anhydrous Na_2_SO_4_, filtered and solvent evaporated. The intermediate product was purified by column chromatography using MeOH:TEA:DCM (v:v:v; 0.5:1:23.5) eluent. Afterwards, it was extracted with ethyl acetate and water. The organic layer was dried over anhydrous Na_2_SO_4_, filtered and solvent evaporated. The obtained product was precipitated from toluene into 15-fold excess of hexane. The precipitate was filtered off and washed with hexane to collect **CO3** as a dark red solid (0.16 g, 15% yield). ^1^H NMR (400 MHz, DMSO-*d*_6_) δ: 8.20 (s, 1H); 8.03 (d, *J* = 8.5 Hz, 2H); 7.11 (d, *J* = 8.5 Hz, 2H); 6.93 (d, *J* = 8.4 Hz, 4H); 6.88 (d, *J* = 8.4 Hz, 4H); 6.66 (d, *J* = 8.3 Hz, 4H); 6.37 (d, *J* = 8.3 Hz, 4H); 5.81 (s, 2H); 3.82 (s, 6H); 3.69 (s, 6H) ppm. ^13^C NMR (101 MHz, DMSO-*d*_6_) δ: 164.15; 159.07; 158.82; 153.44; 149.55; 133.07; 132.98; 132.90; 131.40; 130.09; 128.63; 125.18; 123.86; 117.07; 115.76; 114.17; 113.17; 98.11; 55.34; 55.09 ppm. Anal. calcd for C_42_H_36_N_2_O_6_: C 75.89; H 5.46; N 4.21; found: C 75.56; H 5.70; N 4.24. C_42_H_36_N_2_O_6_ [M^+^] exact mass = 664.26, MS (ESI) = 664.27.

**Diethyl (4-(bis(2,2-bis(4-methoxyphenyl)vinyl)amino)phenyl)phosphonate (1)**

Diethyl (4-aminophenyl)phosphonate (0.55 g, 2.40 mmol) was dissolved in toluene (22 mL + volume of the Dean-Stark trap), (+/-)camphor-10-sulphonic acid (0.84 g, 3.60 mmol) was added and the mixture was heated at reflux for 20 minutes. Afterwards, 2,2-bis(4-methoxyphenyl)acetaldehyde (1.54 g, 6.00 mmol) was added, and reflux continued using a Dean-Stark trap. After termination of the reaction (3.5 h, TLC, THF:*n*-hexane, 3:2) the reaction mixture was extracted with ethyl acetate. The organic layer was dried over anhydrous Na_2_SO_4_, filtered and solvent evaporated. The crude product was purified by flash column chromatography using acetone:*n*-hexane (v:v; 6:19) eluent to collect intermediate compound **1** (1.10 g, 65% yield). ^1^H NMR (400 MHz, DMSO-*d*_6_) δ: 7.62 (dd, *J* = 12.5, 8.2 Hz, 2H); 7.09 (d, *J* = 5.4 Hz, 2H); 6.96 – 6.81 (m, 8H); 6.66 (d, *J* = 7.5 Hz, 4H); 6.38 (d, *J* = 7.5 Hz, 4H); 5.74 (s, 2H); 3.98 (q, *J* = 7.3 Hz, 4H); 3.81 (s, 6H); 3.69 (s, 6H); 1.23 (t, *J* = 7.1 Hz, 6H) ppm. ^13^C NMR (101 MHz, DMSO-*d*_6_) δ: 158.94; 158.67; 148.73; 133.18; 132.96 (d_C-P_, *J* = 10.7 Hz); 131.86; 131.53; 130.08; 128.49; 125.49; 120.09; 118.17; 115.62; 115.47; 114.08; 113.15; 61.36 (d_C-P_, *J* = 5.4 Hz); 55.30; 55.07; 16.23 (d_C-P_, *J* = 6.1 Hz) ppm. Anal. calcd for C_42_H_44_NO_7_P: C 71.48; H 6.28; N 1.98; found: C 71.02; H 6.26; N 2.19. C_42_H_44_NO_7_P [M^+^] exact mass = 705.29, MS (ESI) = 704.59.

**(4-(bis(2,2-bis(4-methoxyphenyl)vinyl)amino)phenyl)phosphonic acid (PO1)**

The obtained intermediate compound **1** (0.50 g, 0.71 mmol) was dissolved in dry 1,4-dioxane (21 ml), and 0.94 ml (7.10 mmol) of bromotrimethylsilane was added dropwise under argon protection. The solution was allowed to stand overnight. Afterwards, the solvent was removed by rotary evaporation under reduced pressure, solid residue was dissolved in methanol (40 ml) and distilled water was added dropwise (40 ml), until solution became opaque. The mixture was stirred during the night. The precipitate was filtered off and washed with water to collect **PO1** as a yellow solid (0.35 g, 76% yield). ^1^H NMR (400 MHz, DMSO-*d*_6_) δ: 7.62 (dd, *J* = 12.5, 8.2 Hz, 2H); 7.05 (d, *J* = 5.9 Hz, 2H); 6.98 – 6.81 (m, 8H); 6.66 (d, *J* = 8.3 Hz, 4H); 6.36 (d, *J* = 8.4 Hz, 4H); 5.71 (s, 2H); 3.81 (s, 6H); 3.69 (s, 6H) ppm. ^13^C NMR (101 MHz, DMSO-*d*_6_) δ: 158.90; 158.62; 147.57; 133.29; 132.16 (d_C-P_, *J* = 10.6 Hz); 131.72; 131.45; 130.07; 128.45; 125.79; 115.17; 115.03; 114.10; 113.14; 55.32; 55.07 ppm. Anal. calcd for C_38_H_36_NO_7_P: C 70.25; H 5.59; N 2.16; found: C 70.14; H 5.93; N 2.49. C_38_H_36_NO_7_P [M^+^] exact mass = 649.22, MS (ESI) = 649.40.

**Diethyl (4-(bis(2,2-bis(4-methoxyphenyl)vinyl)amino)benzyl)phosphonate (2)**

Diethyl 4-aminobenzylphosphonate (0.50 g, 2.06 mmol) was dissolved in toluene (20 mL + volume of the Dean-Stark trap), (+/-)camphor-10-sulphonic acid (0.48 g, 2.06 mmol) was added and the mixture was heated at reflux for 20 minutes. Afterwards, 2,2-bis(4-methoxyphenyl)acetaldehyde (1.11 g, 4.32 mmol) was added, and reflux continued using a Dean-Stark trap. After termination of the reaction (4 h, TLC, THF:*n*-hexane, 3:2) the reaction mixture was extracted with ethyl acetate. The organic layer was dried over anhydrous Na_2_SO_4_, filtered and solvent evaporated. The crude product was purified by column chromatography using acetone:*n*-hexane (v:v; 6.5:18.5) eluent to collect intermediate compound **2** (0.56 g, 38% yield). ^1^H NMR (400 MHz, DMSO-*d*_6_) δ: 7.21 (d, *J* = 6.3 Hz, 2H); 6.96 (d, *J* = 8.2 Hz, 2H); 6.93 – 6.84 (m, 8H); 6.65 (d, *J* = 8.4 Hz, 4H); 6.36 (d, *J* = 8.4 Hz, 4H); 5.66 (s, 2H); 3.96 (q, *J* = 7.3 Hz, 4H); 3.80 (s, 6H); 3.69 (s, 6H); 3.17 (d, *J* = 21.1 Hz, 2H); 1.20 (t, *J* = 7.0 Hz, 6H) ppm. ^13^C NMR (101 MHz, DMSO-*d*_6_) δ: 158.75; 158.43; 144.16; 133.63; 131.90; 130.72 (d_C-P_, *J* = 6.4 Hz); 130.18; 130.10; 128.34; 126.18; 125.08; 116.13; 114.00; 113.08; 61.31 (d_C-P_, *J* = 6.4 Hz); 55.29; 55.05; 30.65 (d_C-P_, *J* = 13.0 Hz); 16.25 (d_C-P_, *J* = 5.8 Hz) ppm. Anal. calcd for C_43_H_46_NO_7_P: C 71.75; H 6.44; N 1.95; found: C 71.15; H 6.67; N 2.11. C_43_H_46_NO_7_P [M^+^] exact mass = 719.30, MS (ESI) = 719.40.

**(4-(bis(2,2-bis(4-methoxyphenyl)vinyl)amino)benzyl)phosphonic acid (PO2)**

The obtained intermediate compound **2** (0.50 g, 0.69 mmol) was dissolved in dry 1,4-dioxane (21 ml), and 0.92 ml (6.95 mmol) of bromotrimethylsilane was added dropwise under argon protection. The solution was allowed to stand overnight. Afterwards, the solvent was removed by rotary evaporation under reduced pressure, solid residue was dissolved in methanol (20 ml) and distilled water was added dropwise (8 ml), until solution became opaque. The mixture was stirred during the night. The precipitate was filtered off and washed with water. The obtained product was precipitated from methanol into 15-fold excess of diethyl ether. The precipitate was filtered off and washed with diethyl ether to collect **PO2** as a yellow solid (0.17 g, 37% yield). ^1^H NMR (400 MHz, DMSO-*d*_6_) δ: 7.20 (d, *J* = 8.0 Hz, 2H); 7.00 – 6.81 (m, 10H); 6.64 (d, *J* = 8.3 Hz, 4H); 6.35 (d, *J* = 8.2 Hz, 4H); 5.63 (s, 2H); 3.80 (s, 6H); 3.68 (s, 6H); 2.90 (d, *J* = 21.1 Hz, 2H) ppm. ^13^C NMR (101 MHz, DMSO-*d*_6_) δ: 158.75; 158.41; 143.75; 133.62; 133.11; 131.99; 130.69 (d_C-P_, *J* = 5.8 Hz); 130.09; 128.31; 127.39; 126.23; 115.94; 114.03; 113.08; 55.30; 55.05; 25.14 ppm. Anal. calcd for C_39_H_38_NO_7_P: C 70.58; H 5.77; N 2.11; found: C 70.21; H 6.05; N 2.17. C_39_H_38_NO_7_P [M^+^] exact mass = 663.24, MS (ESI) = 663.40.

**Diethyl (*E*)-(2-(4-aminophenyl)-1-cyanovinyl)phosphonate (3)**

To a solution of 4-amino benzaldehyde (2.00 g, 16.50 mmol) and diethyl cyanomethylphosphonate (5.90 ml, 36.30 mmol) in toluene (200 ml), piperidine (3.60 ml, 36.30 mmol) was added with stirring, the reaction was heated at reflux overnight. After termination of the reaction (19 h, TLC, acetone:*n*-hexane, 2:3) the reaction mixture was extracted with ethyl acetate. The organic layer was dried over anhydrous Na_2_SO_4_, filtered and solvent evaporated. The crude product was purified by column chromatography using acetone:TEA:*n*-hexane (v:v:v; 7:1:17) eluent to collect intermediate compound **3** (2.71 g, 59% yield). ^1^H NMR (400 MHz, DMSO-*d*_6_) δ: 7.78 (d, *J* = 8.4 Hz, 2H); 7.66 (d, *J* = 20.7 Hz, 1H); 6.64 (d, *J* = 7.7 Hz, 2H); 6.57 (s, 2H); 4.07 (p, *J* = 14.5, 7.4 Hz, 4H); 1.27 (t, *J* = 7.0 Hz, 6H) ppm. ^13^C NMR (101 MHz, DMSO-*d*_6_) δ: 157.93 (d_C-P_, *J* = 8.0 Hz); 154.59; 133.55; 119.74 (d_C-P_, *J* = 17.8 Hz); 117.40 (d_C-P_, *J* = 11.3 Hz); 113.35; 87.04 (d_C-P_, *J* = 201.8 Hz); 62.48 (d_C-P_, *J* = 5.4 Hz); 16.12 (d_C-P_, *J* = 6.0 Hz) ppm. Anal. calcd for C_13_H_17_N_2_O_3_P: C 55.71; H 6.11; N 10.00; found: C 55.77; H 6.24; N 9.73. C_13_H_17_N_2_O_3_P [M^+^] exact mass = 280.10, MS (ESI) = 280.84.

**Diethyl (*E*)-(2-(4-(bis(2,2-bis(4-methoxyphenyl)vinyl)amino)phenyl)-1-cyanovinyl)phosphonate (4)**

The obtained intermediate compound **3** (0.50 g, 1.78 mmol) was dissolved in toluene (20 mL + volume of the Dean-Stark trap), (+/-)camphor-10-sulphonic acid (0.41 g, 1.78 mmol) was added and the mixture was heated at reflux for 20 minutes. Afterwards, 2,2-bis(4-methoxyphenyl)acetaldehyde (0.96 g, 3.74 mmol) was added, and reflux continued using a Dean-Stark trap. After termination of the reaction (2 h, TLC, THF:*n*-hexane, 2:3) the reaction mixture was extracted with ethyl acetate. The organic layer was dried over anhydrous Na_2_SO_4_, filtered and solvent evaporated. The crude product was purified by column chromatography using THF:*n*-hexane (v:v; 8:17) eluent to collect intermediate compound **4** (0.79 g, 59% yield). ^1^H NMR (400 MHz, DMSO-*d*_6_) δ: 8.01 (d, *J* = 8.5 Hz, 2H); 7.91 (d, *J* = 20.8 Hz, 1H); 7.11 (d, *J* = 8.7 Hz, 2H); 6.93 (d, *J* = 8.4 Hz, 4H); 6.88 (d, *J* = 8.4 Hz, 4H); 6.67 (d, *J* = 8.3 Hz, 4H); 6.37 (d, *J* = 8.3 Hz, 4H); 5.80 (s, 2H); 4.12 (p, *J* = 7.3 Hz, 4H); 3.82 (s, 6H); 3.70 (s, 6H); 1.29 (t, *J* = 7.1 Hz, 6H) ppm. ^13^C NMR (101 MHz, DMSO-*d*_6_) δ: 159.07; 158.82; 132.93 (d_C-P_, *J* = 5.9 Hz); 132.62; 131.86; 131.41; 130.09; 130.04; 128.63; 125.20; 124.89 (d_C-P_, *J* = 18.0 Hz); 116.51 (d_C-P_, *J* = 10.6 Hz); 115.66; 114.17; 113.79; 113.17; 93.26 (d_C-P_, *J* = 198.4 Hz); 62.88 (d_C-P_, *J* = 5.4 Hz); 55.34; 55.10; 16.13 (d_C-P_, *J* = 5.9 Hz) ppm. Anal. calcd for C_45_H_45_N_2_O_7_P: C 71.42; H 5.99; N 3.70; found: C 70.97; H 6.09; N 3.44. C_45_H_45_N_2_O_7_P [M^+^] exact mass = 756.30, MS (ESI) = 756.80.
**(*E*)-(2-(4-(bis(2,2-bis(4-methoxyphenyl)vinyl)amino)phenyl)-1-cyanovinyl)phosphonic acid (PO3)**

The obtained intermediate compound **4** (0.40 g, 0.53 mmol) was dissolved in dry 1,4-dioxane (16 ml), and 0.84 ml (6.34 mmol) of bromotrimethylsilane was added dropwise under argon protection. The solution was allowed to stand overnight. Afterwards, the solvent was removed by rotary evaporation under reduced pressure, solid residue was dissolved in methanol (15 ml) and distilled water was added dropwise (7 ml), until solution became opaque. The mixture was stirred during the night. The precipitate was filtered off and washed with water to collect **PO3** as a dark yellow solid (0.27 g, 73% yield). ^1^H NMR (400 MHz, DMSO-*d*_6_) δ: 7.90 (d, *J* = 8.4 Hz, 2H); 7.72 (d, *J* = 19.4 Hz, 1H); 7.08 (d, *J* = 8.4 Hz, 2H); 6.90 (q, *J* = 8.4 Hz, 8H); 6.65 (d, *J* = 8.3 Hz, 4H); 6.37 (d, *J* = 8.2 Hz, 4H); 5.78 (s, 2H); 3.81 (s, 6H); 3.69 (s, 6H) ppm. ^13^C NMR (101 MHz, DMSO-*d*_6_) δ: 158.99; 158.71; 148.35 (d, *J* = 6.6 Hz); 137.04; 133.09; 132.30; 131.52; 130.11; 128.57; 125.91 (d, *J* = 3.6 Hz); 125.38; 123.48; 121.14; 117.83 (d, *J* = 11.3 Hz); 115.79; 114.13; 113.14; 55.33; 55.08 ppm. Anal. calcd for C_41_H_37_N_2_O_7_P: C 70.28; H 5.32; N 4.00; found: C 69.85; H 5.70; N 4.33. C_41_H_37_N_2_O_7_P [M^+^] exact mass = 700.23, MS (ESI) = 699.27.

**Perovskite Solar Cells: Device Fabrication**

**Materials**

Formamidinium iodide (FAI, 99.99%), methylammonium chloride (MACl 99.99%) and methylammonium iodide (MAI) were purchased from Greatcell Solar. Lead (II) iodide (PbI_2_ 99.9%) was purchased from Tokyo Chemical Industry Co., Ltd. (TCI). Cesium iodide (CsI), lead bromide (PbBr_2_), lead (II) chloride (PbCl_2_ 99.9%) and 1,3-diaminopropane dihydroiodide (PDADI) were purchased Xi'an Polymer Light Technology Corp. Pre-patterned ITO substrates were purchased from Advanced Election Technology CO., Ltd. FAPbI_3_, MAPbI_3_, CsPbI_3_, Me-4PACz and 2PACz were purchased from Zhoushan Huazhou Chemical Ltd. Isopropanol (IPA, 99.9%), chlorobenzene (CB, 99.9%), *N,N*-dimethyl formamide (DMF, 99.8%), pure ethanol (EtOH) and dimethyl sulfoxide (DMSO, 99.8%) were purchased from Sigma-Aldrich. Ethyl acetate (EA) was purchased from Macklin.

**Precursor Preparation**

For typical bandgap (1.55 eV) perovskite, a 1.6 mol/L perovskite precursor solution with a composition of Cs_0.05_(FA_0.95_MA_0.05_)_0.95_Pb(I_0.95_Br_0.05_)_3_ was prepared by dissolving stoichiometric amounts of FAI, PbI_2_, MABr, PbBr_2_ and CsI and 10 % MACl in a mixed anhydrous solvent of DMF and DMSO (volume ratio 4:1). For high-efficiency devices, presynthesized CsPbI_3_, FAPbI_3_ and MAPbI_3_ replace CsI, FAI, MAI and PbI_2_ in precursor solution.

For wide bandgap (1.68 eV) perovskite, a 1.5 mol/L perovskite precursor solution with a composition of Cs_0.25_FA_0.75_Pb(I_0.8_Br_0.2_)_3_ was prepared by dissolving stoichiometric amounts of FAI, PbI_2_, PbBr_2_ and CsI and 3% MAPbCl_3_ (mixed by MACl and PbCl_2_) in a mixed anhydrous solvent of DMF and DMSO (volume ratio 4:1). All of the solutions were filtered by 0.22 μm filters before use.

**Device fabrication**

Pre-patterned ITO glass substrates were cleaned with deionized water, anhydrous alcohol and acetone using an ultrasonic cleaner. Then the cleaned ITO substrates were dried and treated with oxygen plasma for 5 minutes prior to use. The solutions of SAM materials (Me-4PACz, 2PACz or enamine-based SAMs) were dissolved in ethanol with a concentration of 1 mmol/L. The solution was spin-coated onto the ITO substrates at 3000 rpm for 30 seconds, followed by annealing at 100 °C for 10 minutes. Subsequently, 50 μL of perovskite precursor solutions were spin-coated at 5000 rpm for 40 s in a nitrogen glove box. During the spin-coating process, ~200 μL EA was rapidly dropped onto the substrates at 30 s, followed by annealing at 100 ℃ for 30 min. The PDADI solution (0.5 mg/mL in IPA) was then spin-coated onto the perovskite layer at 5000 rpm for 30 seconds and annealed at 100 °C for 3 minutes. Finally, 25 nm layer of C_60_, 7 nm layer of BCP, and 120 nm Ag were thermally evaporated in a high-vacuum chamber (< 5 × 10^-4^ Pa) through a metal shadow mask with an aperture area of 0.04 cm^2^.

For mini-module fabrication, the perovskite solar modules consisting of 8 sub-cells connected in series were fabricated on ITO glass substrates. All three scribes (P1, P2, P3) were made with the near-infrared 1064 nm 20 W laser. 6.5 cm × 7 cm ITO substrates were patterned by a laser with a scribing width of 40 μm. The ITO substrates were cleaned and treated with plasma. The following fabrication process of SAM, perovskite, C_60_ and BCP was the same as that of small area devices. Then the substrates were laser scribed over a width of 400 μm. Finally, 80 nm Ag electrode was deposited by thermal evaporation and scribed by a laser with a width of 50 μm. The designed geometric fill factor of the modules was ~92% with an aperture area (AP) of 29.7 cm^2^.

**Characterization and measurements**

XPS characterizations were performed by using Thermo Fisher ESCALAB Xi+ (Al Kα source). XRD patterns were measured by using a Rigaku Smartlab 9KW X-ray diffractometer. SEM images were obtained by using FEI Apreo S LoVac. Atomic force microscope (AFM) and Kelvin probe force microscopy (KPFM) were conducted by Bruker Dimension Icon instrument. PL mapping was obtained by Confocal Microscopy (SPCM-1000) supported by Enli Technology Co., Ltd. Steady-state PL spectra were tested by using Edinburgh FLS1000. The *J-V* characteristics of photovoltaic devices were measured with a solar simulator (Enli Technology) and Keithley 2400 source meter under AM 1.5 G standard irradiation (1000 W/m^2^). Intensity of the solar simulator was calibrated using a certified monocrystalline silicon solar cell (KG5). The active area of device is 0.04 cm^2^, defined by a metal mask. Both reverse scans and forward scans were conducted. External quantum efficiency (EQE) is recorded on a solar cell quantum efficiency measurement system (QE-R) supported by Enli Technology. A calibrated Si diode with a known spectral response is used as a reference.

**Computational Details**

**DFT details**

**Isolated molecule calculations**

All calculations are based on density functional theory (DFT) with Gaussian and ORCA package. Geometries optimization and energy analyses were performed based on the B3LYP functional with Grimme's D3 (BJ) dispersion correction in conjunction with def2-TZVP basis set. The wave function analyses were finished via the Multiwfn 3.8(dev) code and Visual Molecular Dynamics (VMD) visualization program. ^[6,7]^

**Slab DFT calculations**

Since ITO is a doped structure, the concentration of Sn is very low compared to In and O (In_15_SnO_24_) and this would cause an immense number of atoms in a unitcell, with at most 1 or two Sn which hardly changes anything. Usually in publications, people consider In_2_O_3_ instead. So did we and we cleave 111 as this is also the thermally most stable facet. ^[8,9]^

We used Quantum ESPRESSO package^[10]^ using a plane-wave basis set, the generalized gradient approximation of Perdew–Burke–Ernzerhof (PBE)^[11]^ and the Grimme D3^[12]^ dispersion correction. Ultrasoft pseudopotentials were employed with kinetic energy cutoffs of 47 and 323 Ry for the wavefunctions and charge density, respectively. Brillouin-zone integration was carried out using Γ-point-only sampling. Molecular structures were visualized using VMD.^[13]^ The adsorption (binding) energy of the SAM on the ITO surface was calculated as

$$\left[ E_{\text{bind}}=E_{\text{ITO+SAM}}-E_{\text{ITO}}-E_{\text{SAM}} \right]$$

where ($E_{\text{ITO+SAM}}$), ($E_{\text{ITO}}$), and ($E_{\text{SAM}}$) are the total energies of the SAM-adsorbed ITO slab, the bare ITO slab, and the isolated SAM reference, respectively. All energies were computed at the same level of theory (PBE+D3) using structurally relaxed geometries. Negative values of ($E_{\text{bind}}$) indicate favorable (exothermic) binding of the SAM to the surface. For the bidentate anchoring mode, the reference state ($E_{\text{SAM}}$) corresponds to the singly deprotonated phosphonate in the gas phase.

**Molecular Dynamics details**

For the molecular dynamics (MD) simulations, a slab model of the ITO substrate was constructed using experimental lattice parameters, solvated in water at a density of 1.000 g/cm³. Several initial configurations of the SAM molecules were prepared, including oriented and randomly distributed arrangements, to sample different adsorption pathways. Interatomic interactions were modeled using the General AMBER Force Field (GAFF) for the SAM molecules and Buckingham potentials for the ITO substrate,^[14]^ with Lennard-Jones 12/6 potentials governing the SAM-ITO interface. All simulations were performed using the LAMMPS package^[15]^ within a periodic box of dimensions 40 × 40 × 30 Å³. A 6 Å vacuum layer was introduced normal to the surface to mitigate periodic image interactions. Following energy minimization via the steepest descent algorithm (convergence criterion: 10⁻¹⁰ eV/Å), the system was equilibrated and propagated in the NVT ensemble for 500 ps at 300 K using the Nosé–Hoover thermostat with a 1 fs timestep. A cutoff of 8–10 Å was applied for short-range interactions, while long-range electrostatic forces were handled using the PPPM solver with a tolerance of 10⁻⁴. The presence of fixed boundary conditions and the slab geometry necessitated a modified Ewald summation in the z-direction, implemented with a volume factor of 3.

**Figure S1.** Electrostatic potential (ESP) maps on van der Waals (vdW) surface (0.001 a.u. isosurface) of enamine-based SAMs. The color bar is set as blue-white-red for easy observation.

**Figure S2.** ESP and molecular orbitals maps of (a) Me-4PACz and (b) 2PACz.

**Figure S3.** Visualization of the highest occupied molecular orbitals (HOMO) and the lowest unoccupied molecular orbitals (LUMO) of enamine-based SAMs.

**Figure S4.** (a) Chemical structures and average local ionization energy (ALIE) maps on Van der Waals surface of aromatic fragments and SAMs. Yellow spheres indicate position of minima of ALIE on this surface. (b) The minimal value of ALIE of enamine-based SAMs, aromatic fragments and SAMs. The minimal value of ALIE of enamine-based SAMs are listed here: **CO1** 8.41 eV, **CO2** 8.34 eV, **CO3** 8.64 eV, **PO1** 8.36 eV, **PO2** 8.08 eV, **PO3** 8.50 eV.

**Figure S5.** The isosurface maps of Fukui function (*f ^-^*) of 2PACz, Me-4PACz and enamine-based SAMs.

**Figure S6.** Interaction region indicator (IRI) maps of enamine-based SAMs with isovalue of 1.0 a.u. The scale of color bar is given in a.u.

**Figure S7.** IRI maps of enamine-based fragments and V1036 with isovalue of 1.0 a.u.

**Figure S8.** Color-filled maps of electron localization function (ELF) of anchoring groups on benzene rigs in enamine-based SAMs. The atoms noncoplanar with benzene are not shown.

**Figure S9** (a) 3D structure of **PO3** and 2PACz SAM molecules. Independent gradient model based on Hirshfeld partition (IGMH) maps of 2PACz (b) dimer and (c) trimer with isovalue of 0.001 a.u.

**Figure S10.** Surface height and potential difference of corresponding line of ITO/**CO3** and ITO/**PO3**.

**Figure S11.** CPD distribution maps of ITO/**CO3** and ITO/**PO3**.

**Figure S12.** Water contact angles of differently anchored enamine-based SAMs and MeO-2PACz on ITO.

**Figure S13.** XPS C 1s spectra of perovskite films on ITO/SAM.

**Figure S14.** IGMH maps and topology analyses on electron density based on Atoms in Molecules (AIM) theory for **PO3**/Me-4PACz binding with PbI_2_. (a) **PO3** binds with PbI_2_ via methoxy + benzene. (b) **PO3** binds with PbI_2_ via benzene + benzene. (c) Me-4PACz binds with PbI_2_ via carbazole. The isosurface of IGMH is 0.001 a.u. Yellow lines are bond paths, and purple spheres on yellow lines correspond to the (3,-1) critical points (i.e., bond critical point, BCP).

Notes: **PO3** binds with PbI_2_ via electron-rich fragments, including methoxy-benzene or two benzene rings. Me-4PACz doesn’t have methoxy groups and can only binds with PbI_2_ via the electron-rich carbazole ring.

**Figure S15.** Top view SEM images of the perovskite film deposited on ITO/Me-4PACz.

**Figure S16.** SEM images of the bottom interface of the perovskite films deposited on ITO/SAM.

**Figure S17.** The XRD patterns of buried interface of perovskite films. The perovskite films are peeled off by UV glue.

**Figure S18.** (a) Time-resolved photoluminescence (TRPL) spectra. Parameters of TRPL spectra are fitted by two phase exponential decay function (ExpDec2). The fitting following the formula: y = A_1_*exp(-x/*t*_1_) + A_2_*exp(-x/*t*_2_) + y_0_, where A is amplitude, *t* is a time constant. (b) Electrochemical impedance spectroscopy (EIS) curves. (c) Transient photovoltage (TPV) and (d) transient photocurrent (TPC) responses.

**Figure S19.** PL mapping images on a 40 μm × 40 μm region of perovskite films deposited on ITO/SAM, including PL intensity, wavelength and full width at half maximum (FWHM).

**Figure S20.** (a,b) The statistical distribution of *Voc* and FF. The concentration of SAM is 1 mmol/L. (c,d) The statistical distribution of *Voc* and FF. The concentration of SAM is 0.5 mmol/L. (e) *J-V* curves of the devices based on 1.55 eV perovskite (typical bandgap). The concentration of SAM is 0.5 mmol/L.

**Figure S21.** *J-V* curves of Me-4PACz based devices under reverse and forward scan.

**Figure S22.** EQE curves of Me-4PACz, **CO3** and **PO3** devices.

**Figure S23.** *J-V* curves of the champion devices under reverse and forward scan.

**Figure S24.** *J-V* curves of the devices based on 1.68 eV perovskite (wide bandgap).

**Figure S25.** ^1^H and ^13^C NMR spectra of **CO1**.

**Figure S26.** ^1^H and ^13^C NMR spectra of **CO2**.

**Figure S27.** ^1^H and ^13^C NMR spectra of **CO3**.

**Figure S28.** ^1^H and ^13^C NMR spectra of **PO1**.

**Figure S29.** ^1^H and ^13^C NMR spectra of **PO2**.

**Figure S30.** ^1^H and ^13^C NMR spectra of **PO3**.

**Figure S31.** ESI-MS spectrum of **CO1** in wide mass range.

**Figure S32.** ESI-MS spectrum of **CO2** in wide mass range.

**Figure S33.** ESI-MS spectrum of **CO3** in wide mass range.

**Figure S34.** ESI-MS spectrum of **PO1** in wide mass range.

**Figure S35.** ESI-MS spectrum of **PO2** in wide mass range.

**Figure S36.** ESI-MS spectrum of **PO3** in wide mass range.

**Table S1.** Photovoltaic parameters of 1.55 eV champion PSCs on enamine-based SAMs under reverse scan. The concentration of SAM is 0.5 mmol/L.

|  | *Voc* (V) | PCE (%) | FF (%) | *Jsc* (mA cm^-2^) |
| --- | --- | --- | --- | --- |
| **CO1** | 1.122 | 24.08 | 83.18 | 25.80 |
| **CO2** | 1.103 | 23.33 | 82.13 | 25.76 |
| **CO3** | 1.135 | 24.13 | 82.59 | 25.75 |
| **PO1** | 1.165 | 24.34 | 81.05 | 25.78 |
| **PO2** | 1.139 | 23.83 | 81.11 | 25.79 |
| **PO3** | 1.157 | 23.88 | 80.13 | 25.76 |

**Table S2.** Photovoltaic parameters of 1.68 eV champion PSCs under reverse scan.

|  | *Voc* (V) | PCE (%) | FF (%) | *Jsc* (mA cm^-2^) |
| --- | --- | --- | --- | --- |
| 2PACz | 1.112 | 19.33 | 78.88 | 22.04 |
| **PO3** | 1.234 | 22.36 | 80.47 | 22.52 |
| **CO3** | 1.232 | 21.98 | 79.74 | 22.38 |

**REFERENCES**

1.	Vaitukaityte D, Momblona C, Rakstys K, et al. Cut from the Same Cloth: Enamine-Derived Spirobifluorenes as Hole Transporters for Perovskite Solar Cells. *Chemistry of Materials*. 2021;33(15):6059-6067. <https://doi.org/10.1021/acs.chemmater.1c01486>

2.	Miyamoto E, Yamaguchi Y, Yokoyama M. Electrophotography. *Electrophotography*. 1989;28:364.

3.	Cordona M, Ley L. *Photoemission in Solids I*. 1978;26. eISBN 978-3-540-35895-4. https://doi.org/10.1007/3-540-08685-4

4.	Magomedov A, Paek S, Gratia P, et al. Diphenylamine-Substituted Carbazole-Based Hole Transporting Materials for Perovskite Solar Cells: Influence of Isomeric Derivatives. *Advanced Functional Materials*. 2018;28(9):1704351. https://doi.org/10.1002/adfm.201704351

5.	Manivannan R, Satheshkumar A, Elango KP. Highly selective colorimetric/fluorometric chemodosimeters for cyanide ions in aqueous solution based on Michael addition to C-atom possessing different polar substituents. *Tetrahedron Letters*. 2014;55(45):6281-6285. <https://doi.org/10.1016/j.tetlet.2014.09.096>

6.	Lu T, Chen F. Multiwfn: A multifunctional wavefunction analyzer. *Journal of Computational Chemistry*. 2012;33(5):580-592. https://doi.org/10.1002/jcc.22885

7.	Lu T. Visualization Analysis of Covalent and Noncovalent Interactions in Real Space. *Angewandte Chemie - International Edition*. 2025;64(29):e202504895. <https://doi.org/10.1002/anie.202504895>

8.	Zhang, M., Wang, W. and Chen, Y., 2018. Insight of DFT and ab initio atomistic thermodynamics on the surface stability and morphology of In2O3. *Applied Surface Science*, *434*, pp.1344-1352.

9.	Wagner, M., Calcinelli, F., Jeindl, A., Schmid, M., Hofmann, O.T. and Diebold, U., 2022. Adsorption configurations of Co-phthalocyanine on In2O3 (111). Surface Science, 722, p.122065.

10.	P. Giannozzi et al., J. Phys.: Condens. Matter 29, 465901 (2017).
<https://doi.org/10.1088/1361-648X/aa8f79>

11.	J. P. Perdew, K. Burke, and M. Ernzerhof, Phys. Rev. Lett. 77, 3865 (1996).
<https://doi.org/10.1103/PhysRevLett.77.3865>

12.	S. Grimme, J. Antony, S. Ehrlich, and H. Krieg, J. Chem. Phys. 132, 154104 (2010).
<https://doi.org/10.1063/1.3382344>

13.	W. Humphrey, A. Dalke, and K. Schulten, J. Mol. Graphics 14, 33 (1996).
<https://doi.org/10.1016/0263-7855(96)00018-5>

14.	Walsh A, Catlow CRA, Sokol AA and Woodley SM. Physical properties, intrinsic defects, and phase stability of indium sesquioxide. *Chemistry of Materials*. 2009;*21*(20):4962-4969. <https://doi.org/10.1021/cm902280z>

15.	Thompson AP, Aktulga HM, Berger R, et al. LAMMPS-a flexible simulation tool for particle-based materials modeling at the atomic, meso, and continuum scales. *Computer physics communications*. 2022;271:108171. https://doi.org/10.1016/j.cpc.2021.108171
